# Exploring Allosteric Signaling in the Exit Tunnel of the Bacterial Ribosome by Molecular Dynamics Simulations and Residue Network Model

**DOI:** 10.3389/fmolb.2020.586075

**Published:** 2020-09-25

**Authors:** Pelin Guzel, Hatice Zeynep Yildirim, Merve Yuce, Ozge Kurkcuoglu

**Affiliations:** ^1^Department of Chemical Engineering, Istanbul Technical University, Istanbul, Turkey; ^2^Science and Advanced Technology Research and Application Center, Istanbul Medeniyet University, Istanbul, Turkey; ^3^Polymer Research Center and Graduate Program in Computational Science and Engineering, Bogazici University, Istanbul, Turkey

**Keywords:** bacterial ribosome, ribosomal tunnel, allostery, signal relay, trigger factor, translation arrest, antibiotics

## Abstract

The bacterial ribosomal tunnel is equipped with numerous sites highly sensitive to the course of the translation process. This study investigates allosteric pathways linking distant functional sites that collaboratively play a role either in translation regulation or recruitment of chaperones. We apply perturbation response scanning (PRS) analysis to 700 ns long and 500 ns long coarse-grained molecular dynamics simulations of *E. coli* and *T. thermophilus* large subunits, respectively, to reveal nucleotides/residues with the ability to transmit perturbations by dynamic rationale. We also use the residue network model with the *k*-shortest pathways method to calculate suboptimal pathways based on the contact topology of the ribosomal tunnel of *E. coli* crystal structure and 101 ClustENM generated conformers of *T. thermophilus* large subunit. In the upper part of the tunnel, results suggest that A2062 and A2451 can communicate in both directions for translation stalling, mostly through dynamically coupled C2063, C2064, and A2450. For a similar purpose, U2585 and U2586 are coupled with A2062, while they are also sensitive to uL4 and uL22 at the constriction region through two different pathways at the opposite sides of the tunnel wall. In addition, the constriction region communicates with the chaperone binding site on uL23 at the solvent side but through few nucleotides. Potential allosteric communication pathways between the lower part of the tunnel and chaperone binding site mostly use the flexible loop of uL23, while A1336–G1339 provide a suboptimal pathway. Both species seem to employ similar mechanisms in the long tunnel, where a non-conserved cavity at the bacterial uL23 and 23S rRNA interface is proposed as a novel drug target.

## Introduction

**FIGURE 1:**
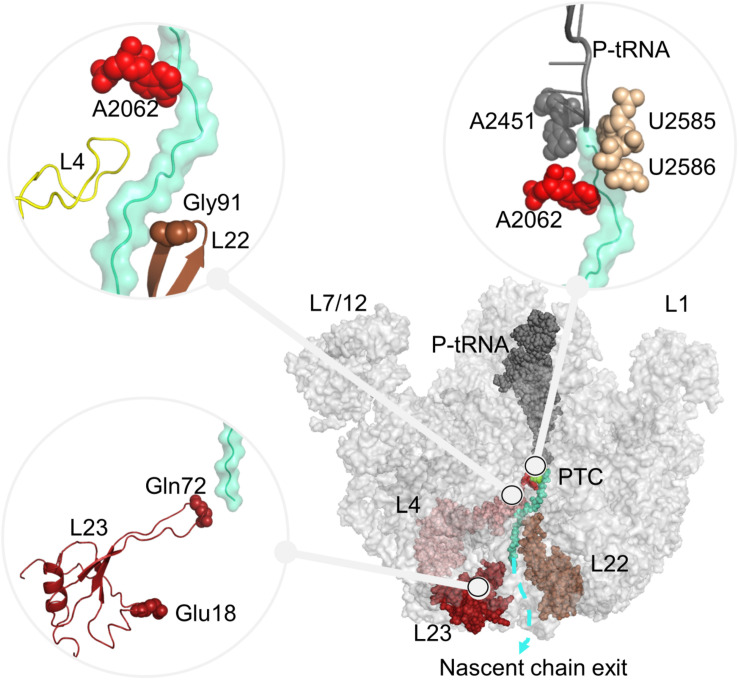
Large subunit 50S, including P-tRNA (gray), polyAla chain (turquoise) and ribosomal proteins protruding to the ribosomal tunnel, namely uL4 (pink), uL22 (brown), uL23 (red) are shown. Nucleotides A2451 (PTC), A2062, U2585–U2586 (ribosomal tunnel), and residues Gly91 (tip of uL22 loop), Glu18 (trigger factor binding site on uL23), Gln72 (tip of uL23 loop), which are investigated in this study are also indicated. In all figures, PyMol (DeLano Scientific LLC., 2002) is used for the molecular visualization.

Ribosomal complexes synthesize proteins according to the genetic information on mRNA across all kingdoms of life. The ribosome complex called as 70S in bacteria is formed by the association of two subunits, small subunit 30S, and large subunit 50S through numerous inter-subunit bridges (Liu and Fredrick, 2016). Each subunit is formed of ribosomal RNAs (16S, 5S, and 23S rRNA) and around 50 ribosomal proteins. The subunits have different functional properties in translation, while they function together as a complex (Ramakrishnan, 2002; Schmeing and Ramakrishnan, 2009). The large subunit 50S catalyzes peptide bond synthesis at the highly conserved catalytic cavity peptidyl transferase center (PTC), where nucleotides G2251, G2252, A2451, C2452, U2506, U2585, A2602 play critical roles in the translation process (Polacek et al., 2003; Youngman et al., 2004; Erlacher et al., 2005; Martin Schmeing et al., 2005; Long et al., 2006; Selmer et al., 2006; Amort et al., 2007; Deutsch, 2014). The nascent polypeptide chain attached to the peptidyl-tRNA (P-tRNA) grows through the ∼100 Å long ribosomal tunnel. The ribosomal tunnel wall is mainly formed of 23S rRNA nucleotides. Nucleotides close to the PTC are highly conserved while nucleotides toward the exit site exhibit variations in bacteria and eukaryotes (Liutkute et al., 2020). Few ribosomal proteins, namely uL4, uL22, and bacteria-specific extension of uL23 also reside on the ribosomal tunnel. The extended loops of these proteins reach from the solvent side into the ribosome exit tunnel as shown in Figure 1. Approximately 25 Å far from the PTC, the loops of uL4 and uL22 form the narrowest part of the ribosomal tunnel, also referred to as the constriction region. Toward to its exit, the ribosomal tunnel accommodates a vestibule, where the long loop of uL23 protrudes.

The ribosomal tunnel is not a passive passageway but is actively taking a role in translation regulation (Wilson and Beckmann, 2011; Ito and Chiba, 2014; Liutkute et al., 2020). Several polypeptides with arrest sequences of up to ∼20 amino acids are known to stall the translation process at the elongation or termination steps for a variety of biological outputs. Some nascent chains require cofactors like amino acids as in TnaC (Cruz-Vera et al., 2005) and antibiotics as in ErmCL (Vazquez-Laslop et al., 2008; Ramu et al., 2011) to stall the protein synthesis in bacteria. Cofactor-dependent translation arrest usually serves to regulate the gene expression related to the cofactor itself. On the other hand, SecM (Yap and Bernstein, 2009; Bhushan et al., 2011) and MifM (Chiba et al., 2009) control their translation without necessitating cofactors. SecM-mediated translation arrest is used to regulate protein export, whereas MifM-mediated translation arrest optimizes both the quality and quantity of membrane proteins under changing physiological conditions. The arrest sequence recognition in these cases realizes due to specific interactions between the nascent chain and the ribosomal constituents at the upper part of the ribosomal tunnel, limited with the PTC and the constriction region. Not far from the PTC, the flexible nucleotide A2062 can trigger a conformational change at the PTC after sensing the arrest sequence on the nascent chain, such as by making contacts with Asp21 on TnaC or Arg163 on SecM, then stall the protein synthesis (Cruz-Vera et al., 2005; Bhushan et al., 2011; Ito and Chiba, 2013). Polypeptide stalling mechanisms also involve direct interactions with nascent chain and the ribosomal tunnel elements A2058, A2059, G2061, A2503, U2504, G2583, U2584, U2585, U2609 (close to the PTC), as well as A751, A752 (close to the constriction region) and flexible loops of uL4 and uL22 (at the constriction region) (Seidelt et al., 2009; Ito and Chiba, 2013; Deutsch, 2014; Figure 1). For the antibiotic-dependent arrest of ErmCL, a signal relay mechanism is suggested between the flexible nucleotide A2062 and nucleotides A2451 and C2452 at the A- site crevice of the PTC, assisted by nucleotides A2503, G2061 and U2504 (Vazquez-Laslop et al., 2008; Ramu et al., 2011). This network of nucleotides is also supported by graph and elastic network studies on *T*. *thermophilus* ribosome complex structures at different translation states (Guzel and Kurkcuoglu, 2017). Similarly, signal relay mechanisms proposed for the SecM include nucleotides A2062 and A2503 (Gumbart et al., 2012) as well as U2585, U2586 and U2506 (Zhang et al., 2015).

Nascent polypeptide chains can compact to adopt secondary structures in the narrower parts of the ribosomal tunnel, and their tertiary structures at the wider regions (Liutkute et al., 2020). Here, the dynamics of the large subunit (Kurkcuoglu et al., 2009), the ribosomal tunnel geometry (Trylska, 2010; Trovato and O’Brien, 2016) together with its electrostatic potential seems to play an important role on complexity and production rate of small folded proteins (Kudva et al., 2018). During its passage through the ribosomal tunnel, compacted chain interacts with the ribosomal tunnel elements and affects the recruitment of chaperones to the exit of the tunnel in bacteria (Trabuco et al., 2010a; Lin et al., 2012; Deutsch, 2014; Denks et al., 2017). This suggests conformational crosstalk not only within the tunnel but also outside the tunnel at the solvent side (Lu et al., 2011; Lin et al., 2012). Here, recruitment of signal recognition particle and trigger factor (TF), both binding uL23 at the solvent side is driven by the nascent chain at the early stages of the translation process. While the nascent chain containing a specific sequence can promote the binding of the signal recognition particle, a compacted nascent chain can lessen the recruitment of the TF to the ribosome complex. These are possibly driven by a network of nucleotides/residues between the extension of uL23 into the ribosomal tunnel and chaperone binding site again on uL23 (Bornemann et al., 2008; Lin et al., 2012) (marked by Glu18 on uL23 in Figure 1). More interestingly, the degree of TF binding is shown to be dependent on the location of the compacted chain in the ribosomal tunnel (Lin et al., 2012). Interactions between the compacted nascent chain and flexible loop of uL23 have a high effect on TF binding, while interactions at the middle parts of the ribosomal tunnel slightly reduce TF recruitment. However, the upper part of the ribosomal tunnel does not affect the recruitment of the chaperone.

Evidently, allostery is an important mechanism at the ribosomal tunnel during translation. The key components that play in regulating the translation process are dispersed along the exit pathway. However, the molecular details of the allosteric communication pathways between these distinct sites remain elusive. At this point, the network of nucleotides and residues on the ribosomal tunnel taking a role in constant communication of the distant functional regions can be considered as targets to eliminate bacterial activity. Indeed, the region marked by the sensor A2062 is an attractive site for macrolides and ketolides in bacteria (Wilson, 2014; Arenz and Wilson, 2016), where most of these antibiotics allosterically stop the catalytic activity of the PTC. To reveal details of allosteric networks and suggest more plausible druggable sites, computational approaches focusing on the contact topology of the ribosomal tunnel can be employed for relatively fast and efficient screening.

In this study, we use two different methods to reveal potential allosteric communication pathways along the ribosomal tunnel: coarse-grained molecular dynamics simulations (Górecki et al., 2009) and residue network model (Guzel and Kurkcuoglu, 2017). Previous coarse-grained molecular dynamics simulations of length 500 ns (Trylska et al., 2005) enabled to observe functional motions of the ribosomal complex. Here, 700 ns long coarse-grained molecular dynamics simulations of the ribosomal complex of *E. coli* with a polyAla chain in the ribosomal tunnel (PDB ID 4v5h; Seidelt et al., 2009) is performed. Then, the perturbation response scanning (PRS) method (Bakan et al., 2011; General et al., 2014) is applied to the resulting covariance matrix to identify effectors and sensors at the ribosomal tunnel. We also calculate *k*-shortest pathways on the residue network representation of ribosomal complex of *E. coli* (PDB ID 4v5h). To reveal any similarities in potential allosteric communication pathways between bacterial species, the ribosomal complex of *T. thermophilus* (PDB ID: 4v5d) is studied with the PRS using 500 ns long coarse-grained molecular dynamics simulations. Then, *k*-shortest pathways of 101 conformers of the ribosomal complex of *T. thermophilus* previously generated by ClustENM (Kurkcuoglu et al., 2016) using PDB ID 4v9m (Zhou et al., 2013) are calculated. Although the specific interactions between the nascent chain and the ribosomal tunnel are critical in the sequence-dependent arrest of translation, the dynamical traits of nucleotides for this task must strongly rely on the topology of the structure. In this line, we aim to reveal pathways of nucleotides/residues that maintain constant communication through tertiary interactions, which can be commonly used in bacteria to regulate the translation of specific nascent chains or the recruitment of chaperones.

We first assess our computational approach by investigating allosteric communication pathways between the flexible A2062 and the PTC A-site A2451, which is previously studied in detail (Vazquez-Laslop et al., 2008; Ramu et al., 2011). Then, we focus on SecM interacting nucleotides U2585–U2586 (*E. coli* numbering), investigate signal relaying in the upper part of the tunnel and discuss our results in the light of previous studies (Nakatogawa and Ito, 2002; Seidelt et al., 2009; Wilson and Beckmann, 2011; Gumbart et al., 2012; Tsai et al., 2014). Finally, allosteric communication of Glu18 on uL23 with two different sites, namely Gln72 on uL23 (lower part of the tunnel, Figure 1), and Gly91 on uL22 β-hairpin (constriction region of the tunnel, Figure 1) is explored. These two residues represent the distinct zones that are reported to play a role in the recruitment of TF (Lin et al., 2012). Each case is discussed in detail while seeking a consensus of the two different methods. Findings for *E. coli* and *T. thermophilus* are also compared, where a common mechanism for allostery in the bacterial ribosomal tunnel as well as a novel drug binding region is proposed.

## Materials and Methods

To reveal potential allosteric communication pathways between distant functional sites, we use two different approaches and two different species. 700 ns long coarse-grained molecular dynamics simulations of *E. coli* ribosomal complex 70S are employed in PRS analysis. Sensitivity profiles of A2062, U2585–U2586 on 23S rRNA, and Glu18 on uL23 are analyzed to determine nucleotides/residues highly coupled to these functional sites in their dynamics. Then, the *k*-shortest pathways method is used to predict suboptimal pathways between distant functional sites around the ribosomal tunnel of *E. coli*. In addition, 500 ns long coarse-grained molecular dynamics simulations of *T. thermophilus* ribosomal complex 70S are investigated with the PRS method. Then, 101 conformers of the *T. thermophilus* ribosomal complex 70S generated with ClustENM are studied with the *k*-shortest pathways method focusing on the same functional sites. Here, molecular dynamics simulations provide local fluctuations of the ribosomal tunnel elements at a time scale that can reflect experimental B-factors. The residue network model used here takes contact topology of the native structure as a basis and highlights the “wirings” between predetermined sites of the molecular machine using the *k*-shortest pathways method. ClustENM provides an effective sampling around the functionally relevant low-frequency motions and gives distinct and reasonable topologies to investigate with the *k*-shortest pathways method.

### Data Set

The crystal structure of the ribosomal complex of *E. coli* with PDB ID 4v5h of resolution 5.8 Å includes 5S, 16S and 23S rRNAs, around 50 ribosomal proteins, a P-tRNA and polyAla chain in the ribosomal tunnel, as depicted in Figure 1 (Seidelt et al., 2009). The crystal structure of *T. thermophilus* ribosomal complex with PDB ID 4v5d of resolution 3.5 Å contains 70S with A-, P-, E-tRNAs, and mRNA (Voorhees et al., 2009). In addition, large subunit 50S from 101 different conformers of the *T. thermophilus* ribosomal complex (PDB ID 4v9m of resolution 4.0 Å) with elongation factor G previously generated by ClustENM (Kurkcuoglu et al., 2016) are used. ClustENM is an iterative algorithm, which generates plausible full-atom conformers by deformation along with the collective modes of the elastic network model. The generated conformers are then clustered, and a representative conformer from each cluster is energetically minimized in implicit solvent. Obtained conformers are taken as starting structures for another round, and this procedure is repeated for several generations. Construction of several generations of conformers at full atomic scale provides an accurate sampling of large conformational changes of biomolecules in large systems. Ribosomal complex conformers employed in the data set were generated using five low-frequency vibrational modes with two generation cycles, which corresponded to five different classes of structures. These structures include functional conformational states, such as the ratchet-like motion of subunits and correlated motion of the L1 stalk with the E-tRNA, as detailed in Kurkcuoglu et al. (2016).

### Coarse-Grained Molecular Dynamics Simulations

Coarse-grained molecular dynamics (CGMD) simulations are performed using RedMD (Górecki et al., 2009), which is suitable to study ribosome dynamics. The full-atom ribosome complexes 70S with PDB IDs 4v5h and 4v5d, are described as a one-bead model, where pseudo-atoms are located at Cα and P atoms to represent residues and nucleotides, respectively. The total potential energy of the structure is given by,

(1) $$E=E_{1-2}+E_{1-3}+E_{1-4}+E_{b\,p}+E_{n\,o\,n\,\text{-}\,b\,o\,n\,d\,e\,d}$$

The harmonic E_1–2_, E_1–3_, and E_1–4_ account for pseudo-bond, pseudo-angle, and pseudo-dihedral interactions involving two, three, and four successive beads, respectively. *E*_*bp*_ indicates the harmonic interactions between the nucleic acid base-pairs, and *E*_*non–bonded*_ energy term represents the Morse potential to determine non-bonded interaction energy considering anharmonicity as,

(2) $$V\,\left(r\right)=A_{P,C_{\alpha }}\,\left(r_{0}\right)\,{\left[1-e\,x\,p\,\left(-\alpha \,\left(r-r_{0}\right)\right)\right]}^{2}$$

*V(r)* is used for both local and non-local non-bonded interactions. The local terms are calculated within a cut-off distance R_*cut–off*_, which is 12.0 Å for Cα and 20.0 Å for P atoms. For the non-local terms, a cut-off distance of 35.0 Å is taken for all nodes. For local interactions, r_0_ is taken as the equilibrium distance in the native structure, while for non-local interactions it changes according to the node type. A_*P,Cα*_ is an exponential function, which differs for P⋯P, Cα⋯Cα and P⋯Cα interactions and decreases with increasing distance between pseudo-atoms. All parameters used in this study are listed in Supplementary Table 1. In order to account for the solvent-ribosomal complex 70S interactions, Langevin dynamics are applied by adding viscous and random forces to Newton’s equation of motion. Here, for the *E. coli* ribosomal complex 70S, two independent simulations of 700 ns are performed. For the ribosomal structure 70S of *T. thermophilus*, two independent simulations of 500 ns are carried. Prior to simulations, each system is subjected to an energy minimization as implemented in RedMD. Each system is heated from 10 to 300 K, and then production simulations are run at 300 K with a collision frequency of 2 ps^–1^ for Langevin dynamics. RedMD describes a constraint between CCA end of P-tRNA and polyAla chain to fix the polypeptide from one end, where the remaining is allowed to fluctuate in the ribosomal tunnel.

### Perturbation Response Scanning

CGMD simulations are used to reveal the effectors and the sensors in the dynamic large subunit 50S of the ribosome. The effectors propagate signals in response to external perturbations and the sensors have a high propensity to sense signals. These two different dynamic properties of nucleotides/residues can shed light on the allosteric mechanisms in the tunnel region of the supramolecule. We used ProDy to perform PRS analysis on the CGMD trajectories (Bakan et al., 2011). In the PRS module of ProDy, a perturbation (one nucleotide/residue at a time) is applied by employing a 3*N*-dimensional force vector based on Hooke’s law *F* = *H*•Δ*R*. Then, displacements of nucleotides/residues as a response to that perturbation is observed considering the overall network. An *N* × *N* PRS matrix (heat map) is generated to display the influence and sensitivity profiles of nucleotides/residues (Atilgan and Atilgan, 2009; General et al., 2014). The *j*th column of the PRS matrix represents the response of all nucleotides/residues to the perturbation at nucleotide/residue *j*, and the average of this column elements point to the signal transmission potential of nucleotide/residue *j* as a sensor. The *i*th row of the matrix describes the response of *i*th nucleotide/residue to perturbations at all other sites and the average of the elements along the row indicates the potential of that nucleotide/residue acting as a propagator or an effector (Dutta et al., 2015).

### *k*-Shortest Pathways

Structures from the data set are represented as undirected weighted graphs, formed of nodes linked by edges. Here, each node is located at Cα (residue) or P atom (nucleotide). The neighboring nodes are linked by edges, where the edge lengths indicate the strength of interactions. In this line, the length of an edge between a node pair (*i*,*j*) is calculated based on their local interaction strengths or affinity *a*_*ij*_ as,

(3) $$a_{i,j}=\frac{N_{\mathrm{ij}}}{\sqrt{N_{i}.N_{j}}}$$

*N*_*ij*_ is the total number of heavy atom-atom neighboring of the (*i,j*) node pair within a cut-off distance of 4.5 Å. A weighting factor of *N_*i*_.N_*j*_* overcomes any bias due to the different sizes of nucleotides/residues. The edge length between (*i,j*) node pair can be described as the inverse of the interaction strength, $a_{i\,j}^{-1}$. With this approach, the edges representing both bonded and non-bonded interactions have comparable values. Here, the communication capability of a node pair is assumed to be proportional to its interaction strength, and thus strongly interacting nodes are close to each other having the ability to transmit information using conformational changes (Brinda and Vishveshwara, 2005; Chennubhotla and Bahar, 2007; Seeber et al., 2015; Guzel and Kurkcuoglu, 2017).

After constructing the residue network model of the ribosome structure, *k*-shortest pathways between the selected source and sink nodes are calculated using Dijskra’s algorithm (Dijkstra, 1959) and Yen’s algorithm (Yen, 1971). As the network is undirected, the source and sink nodes are interchangeable, i.e., *k*-shortest pathways from the source to the sink are identical to those from the sink to the source. The value of *k* = 20 was previously found sufficient to reveal suboptimal pathways on the ribosome complex at different translation states (Guzel and Kurkcuoglu, 2017). This value is controlled for this study as well, which is discussed later. The length (or cost) of each pathway is determined by summing node-pair edge lengths. As one node may be found on more than one pathway, the occurrences of the nodes are calculated. In this way, suboptimal pathways between two functional sites can be determined; moreover, pathways of nodes with high occurrences can be suggested as potential allosteric pathways.

## Results and Discussion

We aim to explore potential allosteric communication pathways between distant regions at the long exit tunnel, also the nucleotides/residues that form these pathways. For this purpose, we focus on three different sites: (1) A2062 at the upper part of the ribosomal tunnel; (2) U2585–U2586 at the upper part of the ribosomal tunnel; and (3) Glu18 on uL23, which marks the binding region of trigger factor (TF) at the solvent side. We discuss our findings following this sequence of locations, i.e., from the upper part of the tunnel to its lower part toward the polypeptide exit. Results from CGMD simulations and *k*-shortest pathways calculations complement each other by revealing dynamic and topological features of the ribosomal tunnel, respectively. Finally, conservation analysis is carried for uL23 sequences of *H. sapiens* and bacteria to reveal potential druggable regions to stop the bacterial activity.

CGMD simulations of 700 ns long are used to obtain the dynamics of the *E. coli* ribosomal complex including both small and large subunits. Root mean square deviation (rmsd) and energy profiles of two independent runs are given in Supplementary Figure 1A. As Run1 has smaller fluctuations in rmsd, this trajectory is analyzed and reported. Principal component analysis (PCA) of the trajectory is carried using Bio3d (Grant et al., 2006). The variance percentages in the scree plot indicate that the first five PCs describe the half of the motions (Supplementary Figure 2A). Here, the PC-one corresponds to the anti-correlated motions of the uL1 and uL11 stalks, while the rotational motion of the small subunit 30S, similar to the ratchet-like motion is also noted (Supplementary Figure 3A). In the PC-two, uL1 stalk makes an anti-correlated motion with respect to the remaining of the complex, and in the PC-three, the anti-correlated motion of the stalks and the small subunit 30S is depicted. The ratchet rotation of the subunits requires GTP hydrolysis on the elongation factor G for the translocation of tRNAs. However, the ribosome complex is able to do a similar motion during the course of the simulations. Other two PCs also correspond to different functional motions of the ribosome complex required for the translation process. In Supplementary Figure 4, normalized B-factors are displayed for the large subunit 50S, and ribosomal proteins uL4, uL22, uL23, which are investigated in this study. The crystal structure 4v5h lacks experimental B-factors, therefore these values are taken from another crystal structure with PDB ID 4v9d (Dunkle et al., 2011) to assess the findings. The Pearson product correlation is calculated to compare the experimental and calculated fluctuations. Correlation coefficients are found as 0.75 (high amplitude fluctuation of the L1 stalk is excluded), 0.71, 0.59, and 0.58 for 23S rRNA, uL4, uL22, and uL23, respectively, which indicate good agreement of the calculated values with experimental data.

We also perform 500 ns long CGMD simulations of the *T. thermophilus* ribosome complex. The rmsd and energy profiles of two independent runs are shown in Supplementary Figure 1B. The rmsd increases up to 5.0 Å due to the large displacement of bL9 extended to the solvent side. Based on smaller fluctuations in rmsd, Run1 is analyzed in this study. The scree plot for the variance percentages of the PCs is shown in Supplementary Figure 2B, where the first five PCs describe more than half of the motions. With a more focused look, the highly flexible bL9 is noted to dominate the motions in the first PCs (not shown). We then exclude bL9 from the PCA to clearly observe collective motions of the ribosomal complex (Supplementary Figure 3B). Accordingly, the PC-one corresponds to the ratchet-like motion of the subunits where two subunits rotate around the same axis in opposite directions. The PC-two shows the correlated motion of uL1 stalk and E-tRNA and the PC-three corresponds to an anti-correlated motion of the subunits such as to open/close the interface from the A-site. All these motions are critical in different steps of the translation. In Supplementary Figure 5A, normalized B-factors are given for the large subunit 50S, including bL9. The Pearson product correlation between the experimental and calculated fluctuations is determined as 0.76 for the 23S rRNA while excluding very high peaks of the calculated fluctuations. Correlation coefficients for the ribosomal proteins uL4, uL22, and uL23 are found as 0.63, 0.75, and 0.45, respectively, where the trends in both fluctuation curves highly agree (Supplementary Figure 5).

Then, perturbation response scanning (PRS) analysis using the covariance matrix from PCA of the large subunit 50S trajectories is carried to get insights into two groups of residues, “sensors,” and “effectors,” which are both important for long-range signal transmission in allostery. In Supplementary Figure 6, the strongest effectors and sensors in the large subunit of *E. coli* and *T. thermophilus* are given. The strongest effectors, which are the most influential nucleotides/residues, are mostly located in the core regions, where the PTC and the ribosomal tunnel are located. The nucleotides A2062 and U2585–U2586, and the residue Glu18 on uL23, which are chosen as source nodes in *k*-shortest pathways calculations, are determined as moderate effectors in the *E. coli* structure (Supplementary Figure 6A). For the *T. thermophilus* structure, A2062 and C2586 are noted to have moderate effectivity when compared to the remaining of the structure (Supplementary Figure 6C). On the other hand, the sensors that are highly sensitive to external perturbations are located at the periphery sites (Supplementary Figures 6B,D). Here, uL1 and uL11 stalks are highly mobile parts of the large subunit (Supplementary Figures 4A, 5A), they have also high sensitivity. This finding is meaningful in the sense that regulation of critical translation steps including the exit of tRNAs and elongation factor-G turnover during protein synthesis by uL1 and uL11, respectively (Harms et al., 2008; Trabuco et al., 2010b). In addition, the ribosomal protein bL9 in *T. thermophilus* structure, which has a closed conformation in the *E. coli* structure, has high flexibility (Supplementary Figure 5A) and high sensitivity (Supplementary Figure 6D). This finding may have a functional significance since bL9 helps the regulation of stress response protein RelA for the survival of the cell under stress conditions (Pei et al., 2017).

### Potential Allosteric Communication Pathways Between A2062 and the PTC

A2062 is a critical nucleotide that interacts with antibiotics and nascent chains, and its related stalling mechanisms include sensing, interpreting, and relaying of a signal to PTC (Vazquez-Laslop and Mankin, 2014). An allosteric communication mechanism for drug-dependent ribosomal stalling was previously suggested between A2062 and nucleotides A2451 and C2452 at the A-site crevice of the PTC (Vazquez-Laslop et al., 2008; Ramu et al., 2011). Here, we further explore this mechanism by investigating a data set including numerous conformers from long CGMD simulations and ClustENM, while comparing the results for the large subunit 50S of *E. coli* and *T. thermophilus*.

**FIGURE 2:**
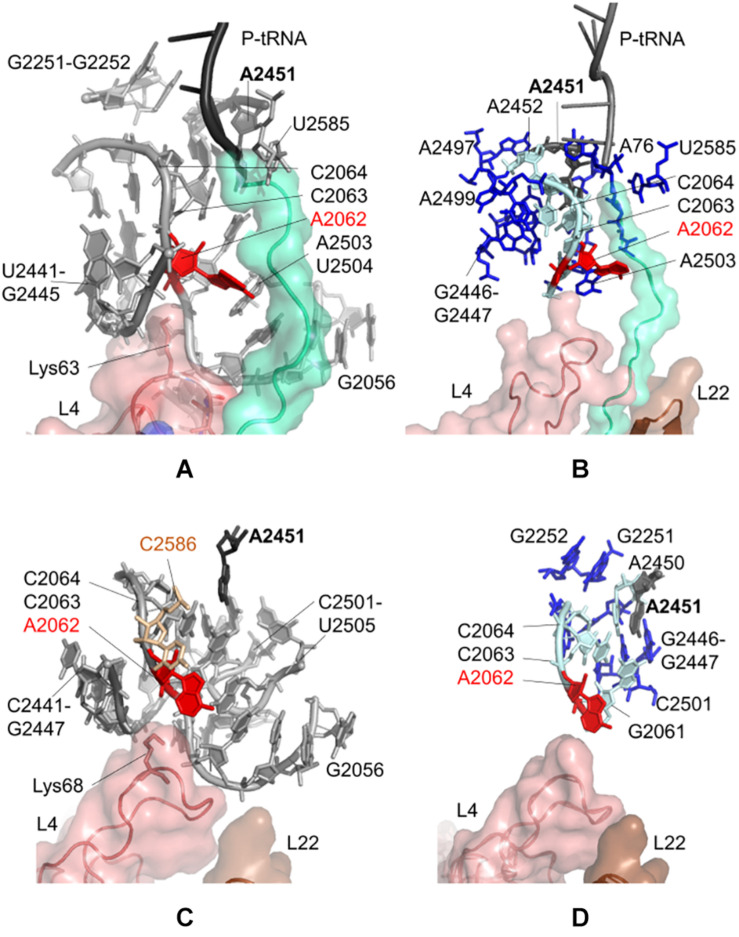
Nucleotides/residues from the sensitivity profile of A2062 based on CGMD simulations of **(A)** *E. coli* ribosomal complex with PDB ID 4v5h, and **(C)** *T. thermophilus* ribosomal complex with PDB ID 4v5d. Nucleotides/residues forming the *k*-shortest pathways on **(B)** *E. coli* large subunit with PDB ID 4v5h, and **(D)** *T. thermophilus* conformers generated by ClustENM using crystal structure with PDB ID 4v9m. polyAla chain in the ribosomal tunnel is shown in turquoise, ribosomal protein uL4 in salmon and uL22 in brown. In **(B,D)** cyan and blue sticks represent the most and the least frequent nucleotides from the calculated pathways, respectively.

Figure 2A indicates locations of the most influential nucleotides on A2062, obtained from the CGMD simulations of *E. coli* ribosomal complex (also listed in Supplementary Table 2). These nucleotides can be classified as having the strongest dynamic coupling with A2062, and thus they have high potential to establish allosteric communication with A2062. Among these, A2450, A2451, A2503, G2061, C2063, C2064, and U2504 are depicted, which are previously proposed to involve in an allosteric network linking the flexible A2062 to the PTC (Ramu et al., 2011; Guzel and Kurkcuoglu, 2017). Here, the universally conserved non-Watson-Crick base-pair A2450–C2063 is highly coupled to A2062, which may help to increase the strength of long-range signal transmission, as was previously suggested (Guzel and Kurkcuoglu, 2017).

A2503 and U2504 are also noted as strongly coupled nucleotides with A2062, underlying their role on allosteric communication, as was previously shown for antibiotic-dependent stalling (Vazquez-Laslop et al., 2008, 2010; Seidelt et al., 2009). The presence of erythromycin restricts the passage of the nascent polypeptide in the tunnel, which in turn forces A2062 to adopt an orientation clashing with A2503. This restriction then stalls the protein synthesis of ErmCL (Vazquez-Laslop et al., 2010). Binding of tiamulin causes similar conformational rearrangements involving A2504 (Gürel et al., 2009). In the CGMD simulations, there is no antibiotic to trigger such a situation, and polyAla chain in the ribosomal tunnel has moderate coupling with A2062, especially from Ala24 and Ala25. Consequently, the coupling of A2062 and A2503–U2504 seems to be inherent to maintain constant communication.

We also note that A2062 and U2585 are coupled, where the latter is in close proximity with Ala24 of the polyAla chain. Indeed, U2585 is known to interact with Pro24 of SecM for ribosomal stalling (Wilson and Beckmann, 2014). In CGMD simulations, Ala24 is sandwiched between U2585 and C2063, which can relay signal from A2062. Another interesting finding is the coupling of A2062 with G2251 and G2252 at the P-site crevice of the PTC. Here, the dynamic coupling is plausibly maintained using the CCA end of P-tRNA and A2450–A2451 at the A-site of the PTC. Another possible route is provided by C2065 and C2066, which are neighboring G2252.

Moreover, sensitivity analysis highlights Lys63–Arg67 on the uL4 loop protruding to the ribosomal tunnel. Especially, the long side chain of Lys63 is oriented to A2062, which suggests the potential role of uL4 in allosteric signaling in this region.

The sensitivity profile of A2451 from CGMD simulations for *E. coli* is also investigated and given in Supplementary Table 3. PRS analysis stresses that the communication between A2451 and A2062 is in both directions; a perturbation on one nucleotide is sensed by the other and vice versa. Similarly, A2451 is also dynamically coupled to G2061–C2066, G2251–G2252 at the P-site crevice of the PTC and G2447–U2448, highlighting these nucleotides as elements of an allosteric network sharing information.

*k* = 20 shortest pathways are calculated between A2062 and A2451 based on the large subunit crystal structure 4v5h of *E. coli.* The cost of the pathways converges for all investigated cases of *E. coli* (Supplementary Figure 7A), indicating that the value of *k* = 20 is suitable for the analysis as was previously shown for the ribosome structures (Guzel and Kurkcuoglu, 2017). The analysis points to nucleotides known to be critical in ribosomal stalling (Figure 2B). The shortest pathway is determined as A2062 → C2063 → A2450 → A2451, where all these nucleotides have a high occurrence in the calculated 20 pathways (Supplementary Table 4 and Supplementary Figure 8A). In addition, four sequential amino acids (Ala21–Ala24) on polyAla chain and A76 of P-tRNA are found on the shortest pathways linking A2062 and A2451, successfully capturing the role of a specific nascent chain – ribosomal tunnel interactions to trigger ribosomal stalling.

CGMD simulations taking a dynamic approach and *k*-shortest pathways method using a static crystal structure have high agreement on allosteric communication pathways at the upper part of the ribosomal tunnel of *E. coli*. Then, we investigate the CGMD simulations of *T. thermophilus* ribosomal complex, lacking the polypeptide chain in the tunnel. PRS analysis suggests that dynamic couplings of nucleotides in the upper part of the ribosomal tunnel (Figure 2C) highly agree with those in the *E. coli* case (Figure 2A), even in the absence of the nascent chain. The lists of nucleotides/residues with high sensitivity values for A2062 and A2451 are given in Supplementary Tables 5, 6, respectively.

In addition, we employ the *k*-shortest pathways method to a collection of 101 large subunit conformers previously generated from the crystal structure 4v9m of *T. thermophilus* using ClustENM (Kurkcuoglu et al., 2016). These conformers are generated around the low-frequency normal modes of the large subunit, which describe global functional motions of the structure, such as anti-correlated motions of the large stalks L1 and L7/L12 (Trylska et al., 2005), and reveal folding zones of the ribosomal tunnel (Kurkcuoglu et al., 2009). Therefore, they provide plausible structures to investigate allosteric communication pathways based on the conformational rearrangements around the ribosomal tunnel. A total of 2020 shortest pathways (*k* = 20 pathways/conformer × 101 conformers) are calculated, where the costs of all pathways converge at *k* = 20 (Supplementary Figure 7B). The analysis indicates that nucleotides with the highest occurrences highly agree with the findings from CGMD and *k*-shortest pathways of the *E. coli* crystal structure (Figure 2D, Supplementary Table 7, and Supplementary Figure 8B). While the nascent chain is missing from the conformers, the shortest pathway is determined as A2062 → C2063 → C2064 → A2450 → A2451. All these nucleotides are commonly determined from CGMD simulations, *k*-shortest pathways calculations of *E. coli*, and *T. thermophilus*. These results imply that signal relay mechanism between two relatively distant functional nucleotides A2062 and A2451 is the same in both species.

The similarity in the findings for both bacterial species stems from the contact topologies of their ribosomal structures. When the large subunits 50S of *E. coli* (4v5h) and *T. thermophilus* (4v9m) are structurally aligned, the rmsd is 2.5 Å over all atoms, and 1.6 Å when only phosphorous atoms are considered. The deviation is due to the flexible uL1 stalk. Then, a cylindrical region with a radius of 40.0 Å around the central axis of the tunnel is taken into account; the rmsd is found as 1.3 Å over all atoms. On the other hand, the rmsd values between the large subunits 50S of *E. coli* (4v5h) and *T. thermophilus* (4v5d) are calculated as 2.3 Å (all atoms), 1.3 Å (only phosphorous atoms), and 0.9 Å (all atoms, tunnel wall). We also calculate the number of contacts of the nucleotides investigated in this study (Supplementary Figure 9). Accordingly, the contact numbers are highly similar for *E. coli* and *T. thermophilus* structures.

Successful prediction of critical residues of the well-known signal relay mechanism at the upper part of the ribosomal tunnel motivates us to employ our approach for estimating allosterically predisposed nucleotides/residues between other distal functional sites in *E. coli* and *T. thermophilus*.

### Potential Allosteric Communication Pathways Between U2585–U2586 and the PTC

**FIGURE 3:**
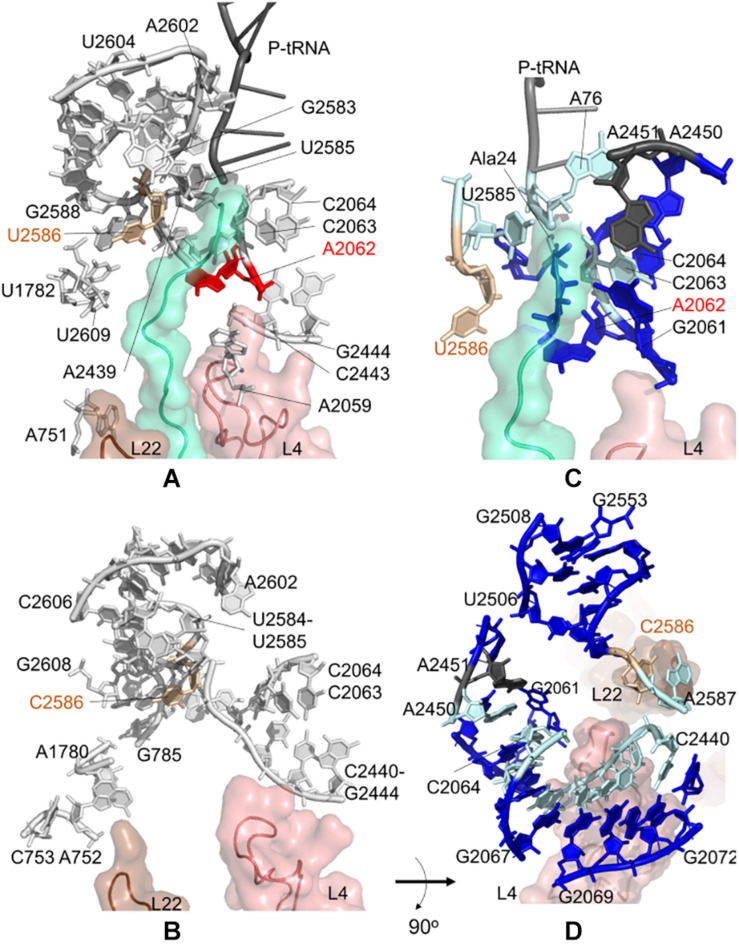
Nucleotides/residues from the sensitivity profile of U2585–U2586 based on CGMD simulations of **(A)** *E. coli* ribosomal complex with PDB ID 4v5h, and **(B)** *T. thermophilus* with PDB ID 4v5d. Nucleotides forming the *k*-shortest pathways on **(C)** *E. coli* large subunit with PDB ID 4v5h, and **(D)** *T. thermophilus* conformers generated by ClustENM using crystal structure with PDB ID 4v9m. Coloring is as in Figure 2.

During the synthesis of SecM, the ribosomal stalling process requires two components: a well-conserved stalling sequence and a ribosomal tunnel topology ready to detect this important detail, where U2585 and U2586 play a critical role (Zhang et al., 2015). Here, we focus on the contact topology of the ribosomal tunnel and investigate the sensitivity profiles for U2585–U2586 obtained from CGMD and PRS analyses. For the *E. coli* case, nucleotides A751–A753, A781–A782, U1782, A2062, A2439, and A2602 are found to be dynamically coupled to U2585–U2586, implying their role on long-range signal transmission between U2585–U2586 and the PTC (Figure 3A and Supplementary Tables 8, 9).

We note two apparent networks of nucleotides coupled to U2585–U2586 dynamics at the opposite sides of the ribosomal tunnel. The first contains U1782, U2609, and A751 neighboring the flexible β-hairpin of uL22. The other involves A2062, C2063, C2064, C2443, G2444, and A2059 next to the uL4 loop. The constriction region of the tunnel, where uL4 and uL22 loops protrude, is therefore linked to U2585–U2586. Moreover, closer to the tunnel entrance, A2439 and A2062 are coupled with U2585–U2586. These three networks of nucleotides agree well with the previous structural study on SecM mediated stalling (Seidelt et al., 2009). The analysis indicates that U2585–U2586 are not coupled with A2450–A2451 at the PTC, to which a signal/perturbation is plausibly directed through A2062–C2064 as previously discussed. As the polypeptide in the ribosomal tunnel does not contain a stalling sequence, we do not detect any significant coupling between the polyAla chain and nucleotides U2585–U2586. At this point, the results underline that at the upper part of the ribosomal tunnel, there exist multiple sites constantly monitoring and communicating during the translation of chains with or without stalling sequences.

Interestingly, we detect the same picture for the *T. thermophilus* ribosome tunnel: three different networks of nucleotides linking U2585–C2586 (i) to A2602 using C2441–C2442, (ii) to uL4 using C2063, C2064, C2443, G2444, and (iii) to uL22 using G785, A1780, A752, C753 (Figure 3B and Supplementary Tables 10, 11).

Closer to the PTC of *E. coli*, A2602 is coupled to U2585 and U2586. A2602 is known to be critical in nascent peptide release (Polacek et al., 2003) but not in drug-dependent translation arrest of ErmCL (Koch et al., 2017). Additionally, in all species, sparsomycin binds A2602 to change the PTC conformation (Porse et al., 1999). We also note the dynamic coupling of A2602 with U2586–C2586 in *T. thermophilus*. Considering the location and role of the highly conserved A2602 in the PTC, this nucleotide has a high potential to take a role in the translation arrest of other nascent chains that can employ different signal relay mechanisms.

Then, *k* = 20 shortest pathways between U2586 and A2451 are determined for *E. coli* large subunit structure (Figure 3C, Supplementary Table 4 and Supplementary Figure 10A). The most frequently occurring nucleotides are determined as U2585, C2063, C2064, and A76 of P-tRNA, which are suggested to maintain distant communication. Here, as the method is based on the contact topology of the structure, residues of the polyAla chain also involve in suboptimal pathways. The shortest pathways are in good agreement with the CGMD results as well as with the previously reported signal relay mechanisms (Seidelt et al., 2009).

Shortest pathways calculations between U2586 and A2451 on *T. thermophilus* large subunit conformers point to C2063–C2066, C2440–G2446, A2450, and A2587 as the nucleotides with highest occurrences (Figure 3D, Supplementary Table 7 and Supplementary Figure 10B). The shortest pathways calculated for these conformers involve more neighboring nucleotides when compared to *k*-shortest pathways for *E. coli* structure, due to lack of polypeptide in the ribosomal tunnel. Nonetheless, CGMD and *k*-shortest pathways of ClustENM conformers agree on the potential allosteric pathways. The contact topology points to functionally important nucleotides, such as G2251 at the P-site of PTC (Supplementary Table 7). Highly conserved flexible U2506 is another important nucleotide found from the calculations. This nucleotide plays a key role in peptide bond synthesis (Martin Schmeing et al., 2005) and contributes to pleuromutilin binding pocket together with A2058, A2059, and G2505 in *E. coli* and *T. thermophilus* (Long et al., 2006; Killeavy et al., 2020).

### Potential Allosteric Communication Pathways Between uL23 and the Ribosomal Tunnel

**FIGURE 4:**
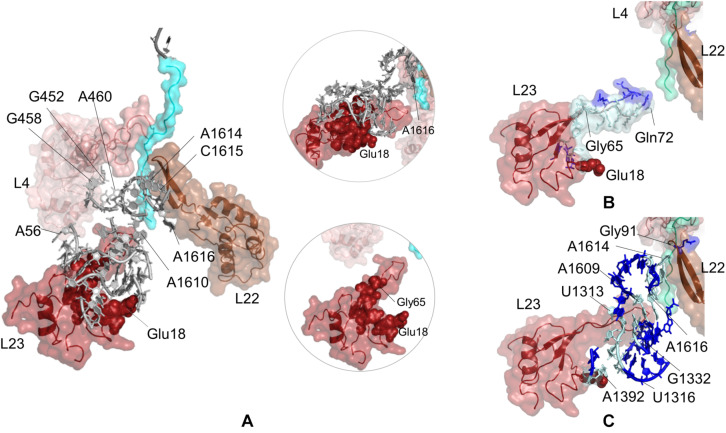
**(A)** Nucleotides/residues from the sensitivity profile of Glu18 (uL23) based on CGMD simulations of *E. coli* ribosomal complex with PDB ID 4v5h. Insets show pathways from different perspectives. Nucleotides/residues forming the *k*-shortest pathways **(B)** between Glu18 (uL23) and Gln72 (uL23) and **(C)** between Glu18 (uL23) and Gly91 (uL22) on *E. coli* large subunit with PDB ID 4v5h. Coloring is as in Figure 2.

The sensitivity profile of Glu18 on uL23 from CGMD simulations is visualized in Figure 4A and given in Supplementary Table 12. Nucleotides/residues dynamically coupled to Glu18 are mostly populated at the lower part of the ribosomal tunnel. On uL23, residues His15–Ser17, Lys33, Val63, Gly65, Lys81, Lys82 are highlighted, where Gly65 and Lys81 are located on the hinge of the flexible loop protruding to the tunnel. Moreover, 23S rRNA nucleotides G1339, G1395, A1610, A1616, have high potential to relay signal at the lower part of the ribosomal tunnel. Here, the polyAla chain from the crystal structure is 20 amino acids long and does not interact with the uL23 loop. Still, our findings highly agree with the experimental observations indicating that the interactions between the compacted nascent chain and the lower part of the tunnel strongly modulate the recruitment of TF and signal recognition particle (Lin et al., 2012).

While nucleotides of 23S rRNA and residues of uL23 are dynamically coupled at the lower part of the tunnel, this coupling seems to continue toward the constriction region of the tunnel through few nucleotides. As noted from Figure 4A, A1610–A1616 on 23S rRNA neighboring β-hairpin of uL22 can plausibly assist relaying signal between the inner wall of the ribosomal tunnel at the constriction region and solvent side. In addition, A56–U59, G452, and G458–A460 of 23S rRNA reaching the hinge of the uL4 loop are also involved in a network of coupled nucleotides linking the constriction region and the chaperone binding site. We do not detect any other nucleotide/residue near the upper regions of the tunnel coupled to Glu18. These findings support the previous FRET results (Lin et al., 2012); the chaperone binding site is weakly linked to the constriction region marked by flexible loops of uL4 and uL22, but not to upper regions close to the PTC. However, if an allosteric communication between the chaperone binding site and the PTC exists, approaches achieving higher time scales would be necessary to reveal the mechanism.

Potential allosteric communication pathways between Glu18 on uL23 and ribosomal tunnel of *E. coli* are further investigated using the *k*-shortest pathways method. Since the method requires a source and a sink node, we first calculate *k* = 20 shortest pathways between Glu18 and Gln72 (uL23). Twenty shortest pathways include only residues of uL23 based on contact topology of the crystal structure (Figure 4B, Supplementary Table 4 and Supplementary Figure 11A). Here, tertiary interactions on uL23 trace a path using His70 (tip of uL23 loop) → Gly65 (hinge of uL23 loop) → Lys64 → Val63 → Asp79 → Trp80 → Lys33 → Ser17 → His15 → Glu18, between the inside of the tunnel and the solvent side, consistent with CGMD results. Then, *k* = 20 shortest pathways are calculated between Glu18 and Gly91 on uL22 β-hairpin (Supplementary Figure 12A and Supplementary Table 4). Gly91 is known to be a hot spot for the nascent chain stalling (Wilson and Beckmann, 2011). As displayed in Figure 4C, the shortest pathways involve mostly 23S rRNA nucleotides, where Lys19 (uL23) → A1392 → U1316 → C1315 → C1314 → G1332 → A1609 → A1616 → C1615 → A1614 is the shortest route between distant Glu18 (uL23) and Gly91 (uL22). These results highly agree with the PRS analysis of CGMD simulations and also suggest the suboptimal pathways between these distant sites.

**FIGURE 5:**
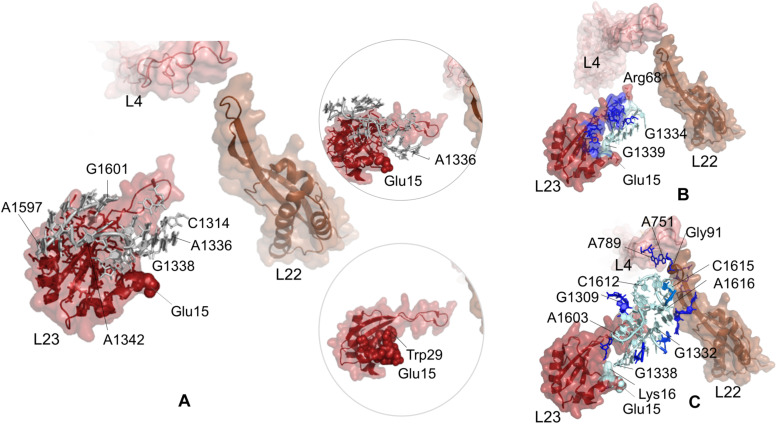
**(A)** Nucleotides/residues from the sensitivity profile of Glu15 (uL23) based on CGMD simulations of *T. thermophilus* ribosomal complex with PDB ID 4v5d. Insets show pathways from different perspectives. Nucleotides/residues forming the *k*-shortest pathways **(B)** between Glu15 (uL23) and Arg68 (uL23) and **(C)** between Glu15 (uL23) and Gly91 (uL22) on ClustENM conformers of the *T. thermophilus* large subunit with PDB ID 4v9m. Coloring is as in Figure 2.

Potential communication pathways between chaperone binding site on uL23 and both lower part and the constriction region of the tunnel are investigated for large subunit 50S of *T. thermophilus*. Figure 5A displays the nucleotides and residues, which are dynamically coupled to Glu15 of uL23. Similar to the findings for *E. coli* simulations using PRS analysis, nucleotides/residues with high sensitivity cluster on and around uL23, but interestingly they do not reach uL22. Here, CGMD simulations of the *T. thermophilus* ribosome complex are 500 ns long, whereas time length is 700 ns for the *E. coli* ribosome complex simulations. This implies that the signal transmission between these distant regions, Glu15 on uL23 and the loop of uL22, plausibly requires longer than 500 ns. Then, ClustENM conformers of *T. thermophilus* are analyzed with the *k*-shortest pathways method. Figure 5B shows the nucleotides and residues on a total of 2020 shortest pathways between Glu15Tt (*T. thermophilus* numbering) and Arg68Tt at the tip of the uL23 loop. Two suboptimal pathways are noted; one tracing uL23 residues similar to the results for *E. coli* (see Figure 4B) and the other using 23S rRNA nucleotides. Shortest pathways calculations point that allosteric communication between chaperone binding site and the lower part of the tunnel can employ tertiary interactions both on uL23 and 23S rRNA depending on the conformational rearrangements (Supplementary Table 7 and Supplementary Figure 11B). It is worth to note here that ClustENM conformers provide conformations that reveal shortest pathways similar to those obtained from PRS analysis of CGMD simulations.

Suboptimal pathways between Glu15 on uL23 and Gly91 on uL22 determined from ClustENM conformers of *T. thermophilus* are shown in Figure 5C (also see Supplementary Table 7 and Supplementary Figure 12B). These pathways employ similar nucleotides as in *E. coli*, where nucleotides G1332Tt-G1338Tt and C1612Tt-A1616Tt are highlighted as potential components of an allosteric network common to both ribosomal structures.

Finally, we perform conservation analysis of ribosomal protein uL23 by multiple sequence alignment for *E. coli*, *T. thermophilus*, and *H. sapiens* to explore suitable sites for drug design. Sequence alignments are done using the Clustal Omega program with the default settings on the UniProt.org server. Supplementary Figure 13A displays the results of the *E. coli* structure. Here, the hinge of the uL23 loop, which plausibly plays a critical role in signal relaying in trigger factor recruitment, is not conserved among human and *E. coli*/*T. thermophilus*. Especially, a small non-conserved cavity is detected around the hinge of the uL23 loop, contoured by residues Lys9, Arg12, His15, Lys33, Lys36, Ser78, and Trp80 in *E. coli*. Functional motions of long loops are often controlled by the hinge regions, highlighting this site attractive as a drug target. Moreover, electropositive side chains of these residues interact with the electronegative backbone of U59–U62 on 23S rRNA, which in turn holds uL23. Binding of a small molecule on this cavity while interacting with the 23S rRNA nucleotides can affect the functional motions of uL23 in the ribosome complex. Worth to note that conservation of the cavity is low among bacteria as well (Supplementary Figure 13B), which in turn suggests this region as a species-specific target site. Moreover, uL23 also hosts the signal recognition particle providing two binding sites; globular domain (Glu18 and Glu52) and the loop (Gly71) (Denks et al., 2017). Accordingly, interacting with the tip of the uL23 loop is suggested to enable the signal recognition particle to sense the arrival of the nascent chain. After sensing the nascent chain from the loop motions, the binding affinity of the chaperone apparently increases, then the chaperone proceeds with the standby or anticipatory mode and later with the recognition step. Consequently, blocking the motions of this loop can also affect the binding of the signal recognition particle.

## Conclusion

The ribosomal tunnel can be considered as having three compartments, an upper part, a middle part and a lower part, similar to folding zones (Deutsch, 2014), where separate control elements regulate translation process. At the upper part, 23S rRNA nucleotides A2062, U2585, U2586 control co-factor dependent/independent translation arrest of specific sequences (Cruz-Vera and Yanofsky, 2014; Ito and Chiba, 2014; Vazquez-Laslop and Mankin, 2014). Our results indicate that, even in the absence of a specific stalling sequence or a co-factor, a network of inherently coupled nucleotides exists, which is ready to detect the sequence then stall the translation. Especially, CGMD simulations point out that the communication of A2062 and A2451 at the PTC is in both directions, dictated by the contact topology. On the other hand, critical nucleotides U2585–U2586 are not dynamically coupled to the PTC, yet they can communicate with A2451 through C2063–C2064. We determine two other distinct suboptimal pathways linking U2585–U2586 to uL4 and uL22 loops at the constriction region, which marks the middle part of the ribosomal tunnel.

At the lower part of the tunnel, other allosteric communication pathways plausibly exist to regulate the recruitment of chaperones to the ribosomal complex. Here, uL23 is acting as a bridge between the chaperone binding region at the solvent side and the vestibule, where compacted chains are waiting to emerge. We suggest that the chaperone binding site is strongly communicating with the ribosomal tunnel using the uL23 loop and His15, Ser17, Lys33, while nucleotides A1336–G1339 also contribute. On the other hand, a weak signal relaying path from the chaperone binding site uses nucleotides G458–A460 and C1611–C1615 respectively reaching uL4 and uL22 loops at the constriction region. Based on 700 ns long CGMD simulations, we do not detect any dynamic coupling between the chaperone binding site and the upper part of the ribosomal tunnel.

As the contact topology of *E. coli* and *T. thermophilus* are highly similar, PRS analysis results and *k*-shortest pathways calculations point to similar suboptimal pathways implying similar signaling mechanisms at their ribosomal tunnels. The conservation analysis of uL23 using *H. sapiens*, *E. coli*, and *T. thermophilus* sequences reveals a non-conserved pocket contoured by polar amino acids as well as 23S rRNA nucleotides U59–U62, which is proposed as a novel site for drug design to disrupt the function of uL23.

## Data Availability Statement

The raw data supporting the conclusions of this article will be made available by the authors, without undue reservation, to any qualified researcher.

## Author Contributions

PG and OK designed the work. PG and HZY carried the molecular dynamics simulations and *k*-shortest pathways calculations, respectively. PG, HZY, MY, and OK analyzed the findings. PG and OK wrote and edited the manuscript. All authors contributed to the article and approved the submitted version.

## Conflict of Interest

The authors declare that the research was conducted in the absence of any commercial or financial relationships that could be construed as a potential conflict of interest.
