# Supplementary material for: Exploring Allosteric Signaling in the Exit Tunnel of the Bacterial Ribosome by Molecular Dynamics Simulations and Residue Network Model
Source: Front Mol Biosci. 2020 Sep 25;7:586075. doi: 10.3389/fmolb.2020.586075 (PMC7545307; doi:10.3389/fmolb.2020.586075)
Supplement: Supplementary file 1 [file Data_Sheet_1.PDF]

Supplementary Material

Exploring allosteric signaling in the exit tunnel of the bacterial ribosome by molecular dynamics simulations and residue network model

Pelin Guzel[1,2], Hatice Zeynep Yildirim[3], Merve Yuce[1], Ozge Kurkcuoglu[1*]

[1]Department of Chemical Engineering, Istanbul Technical University, Istanbul, Turkey

[2]Science and Advanced Technology Research and Application Center, Istanbul Medeniyet University, Istanbul, Turkey

[3]Polymer Research Center and Graduate Program in Computational Science and Engineering, Bogazici University, Istanbul, Turkey

*** Correspondence:**
olevitas@itu.edu.tr

Supplementary Table 1. Force field parameters for RedMD

Parameters	C_α beads	P beads
$k_{1-2}\left[\frac{kcal}{mol.Å^2}\right]$	50.0	3.0
$k_{1-3}\left[\frac{kcal}{mol.Å^2}\right]$	5.0	2.5
$k_{1-4}\left[\frac{kcal}{mol.Å^2}\right]$	3.0	0.5
$k_{bp}\left[\frac{kcal}{mol.Å^2}\right]$	n/a	0.6
α [1/Å]	0.707	0.707
$A_{P,C_\alpha}(r_0)\left[\frac{kcal}{mol.Å}\right]$	$4 \times \exp(-\frac{r_0}{2.8})$	$2 \times \exp(-\frac{r_0}{6})$
$R^{cut-off}$ [Å]	12.0	20.0
$R^{cut-off}_{max}$ [Å]	35.0	35.0

* k_{1-2}, k_{1-3} and k_{1-4} are the force constants of harmonic interactions E_{1-2}, E_{1-3} and E_{1-4} in Equation 1.

Supplementary Table 2. Nucleotides/residues highly influencing A2062 in simulations of *E. coli* 70S

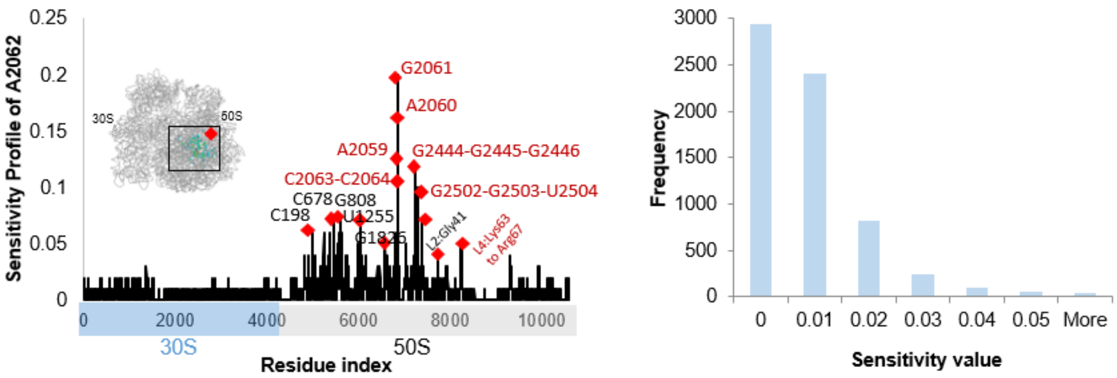

Residue		Sensitivity	Residue		Sensitivity	Residue		Sensitivity	Residue		Sensitivity
23S	449	0.06	23S	1248	0.04	23S	2071	0.06	23S	2498	0.04
23S	452	0.06	23S	1249	0.05	23S	2072	0.05	23S	2499	0.05
23S	585	0.06	23S	1250	0.04	23S	2073	0.05	23S	2500	0.05
23S	739	0.04	23S	1251	0.05	23S	2074	0.04	23S	2501	0.05
23S	747	0.05	23S	1252	0.03	23S	2075	0.04	23S	2502	0.08
23S	748	0.04	23S	1253	0.04	23S	2240	0.04	23S	2503	0.10
23S	773	0.04	23S	1254	0.04	23S	2243	0.05	23S	2504	0.06
23S	778	0.04	23S	1255	0.06	23S	2244	0.04	23S	2505	0.04
23S	779	0.04	23S	1256	0.07	23S	2246	0.04	23S	2585	0.02
23S	780	0.04	23S	1257	0.07	23S	2247	0.05	23S	2586	0.02
23S	782	0.04	23S	1258	0.05	23S	2248	0.04	23S	2587	0.03
23S	783	0.04	23S	1259	0.04	23S	2249	0.05	23S	2588	0.04
23S	784	0.04	23S	1261	0.04	23S	2251	0.04	23S	2589	0.04
23S	787	0.04	23S	2012	0.04	23S	2252	0.04	23S	2590	0.07
23S	791	0.05	23S	2016	0.05	23S	2429	0.04	23S	2591	0.04
23S	792	0.04	23S	2017	0.06	23S	2431	0.04	23S	2592	0.04
23S	793	0.05	23S	2018	0.05	23S	2436	0.04	23S	2593	0.03
23S	794	0.06	23S	2019	0.03	23S	2438	0.05	23S	2599	0.04
23S	795	0.05	23S	2025	0.04	23S	2439	0.05	23S	2600	0.04
23S	796	0.04	23S	2031	0.04	23S	2440	0.05	uL2	42	0.04
23S	797	0.04	23S	2032	0.04	23S	2441	0.06	uL4	56	0.03
23S	798	0.05	23S	2056	0.07	23S	2442	0.07	uL4	63	0.05
23S	799	0.05	23S	2057	0.07	23S	2443	0.10	uL4	64	0.04
23S	800	0.03	23S	2058	0.08	23S	2444	0.12	uL4	65	0.05
23S	801	0.04	23S	2059	0.11	23S	2445	0.10	uL4	66	0.05
23S	802	0.05	23S	2060	0.16	23S	2446	0.10	uL4	68	0.04
23S	803	0.05	23S	2061	0.20	23S	2447	0.05	uL4	73	0.04
23S	804	0.05	23S	2063	0.12	23S	2448	0.08	uL4	76	0.04
23S	805	0.05	23S	2064	0.11	23S	2449	0.06	uL4	77	0.03

Supplementary Table 2. Continued

23S	806	0.07	23S	2065	0.09	23S	2450	0.05	uL4	81	0.04
23S	807	0.07	23S	2066	0.07	23S	2451	0.05	uL4	82	0.05
23S	808	0.06	23S	2067	0.06	23S	2452	0.04	uL15	1125	0.03
23S	809	0.04	23S	2068	0.04	23S	2453	0.04	uL15	1126	0.04
23S	810	0.05	23S	2069	0.07	23S	2496	0.05	uL15	1127	0.04
23S	811	0.05	23S	2070	0.05	23S	2497	0.06			

Supplementary Table 3. Nucleotides/residues highly influencing A2451 in simulations of *E. coli* 70S

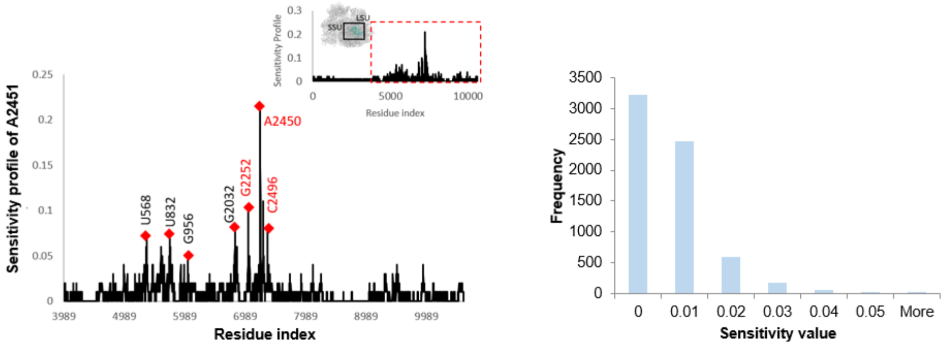

Residue		Sensitivity	Residue		Sensitivity	Residue		Sensitivity	Residue		Sensitivity
23S	193	0.04	23S	948	0.04	23S	2036	0.04	23S	2453	0.11
23S	194	0.04	23S	949	0.04	23S	2055	0.05	23S	2454	0.09
23S	532	0.04	23S	950	0.04	23S	2056	0.05	23S	2455	0.09
23S	533	0.04	23S	953	0.04	23S	2057	0.05	23S	2456	0.06
23S	564	0.04	23S	954	0.04	23S	2059	0.04	23S	2457	0.05
23S	565	0.04	23S	955	0.05	23S	2060	0.05	23S	2458	0.04
23S	566	0.05	23S	956	0.07	23S	2061	0.06	23S	2459	0.05
23S	567	0.04	23S	957	0.05	23S	2062	0.04	23S	2460	0.05
23S	568	0.06	23S	958	0.04	23S	2064	0.04	23S	2492	0.04
23S	569	0.05	23S	961	0.04	23S	2065	0.06	23S	2493	0.04
23S	570	0.07	23S	962	0.05	23S	2066	0.06	23S	2494	0.05
23S	571	0.05	23S	963	0.06	23S	2067	0.06	23S	2495	0.09
23S	572	0.04	23S	964	0.05	23S	2068	0.05	23S	2496	0.11
23S	573	0.06	23S	965	0.04	23S	2069	0.04	23S	2497	0.11
23S	574	0.06	23S	972	0.04	23S	2247	0.04	23S	2498	0.10
23S	575	0.06	23S	973	0.04	23S	2248	0.04	23S	2499	0.09
23S	576	0.06	23S	974	0.04	23S	2249	0.05	23S	2500	0.08
23S	585	0.04	23S	976	0.04	23S	2250	0.06	23S	2501	0.06
23S	671	0.04	23S	983	0.04	23S	2251	0.10	23S	2502	0.05
23S	675	0.04	23S	992	0.04	23S	2252	0.08	23S	2503	0.05
23S	676	0.04	23S	1135	0.04	23S	2253	0.04	23S	2504	0.05
23S	805	0.04	23S	1190	0.04	23S	2260	0.04	23S	2505	0.05
23S	806	0.05	23S	1255	0.04	23S	2264	0.04	23S	2506	0.05
23S	807	0.06	23S	1256	0.05	23S	2265	0.04	23S	2507	0.05
23S	808	0.04	23S	1257	0.04	23S	2275	0.05	23S	2508	0.04
23S	810	0.04	23S	2017	0.04	23S	2276	0.04	23S	2571	0.08
23S	825	0.04	23S	2018	0.04	23S	2277	0.04	23S	2572	0.05
23S	826	0.04	23S	2020	0.04	23S	2428	0.04	23S	2573	0.06
23S	827	0.04	23S	2026	0.04	23S	2429	0.04	23S	2574	0.05
23S	828	0.05	23S	2027	0.04	23S	2444	0.04	23S	2575	0.05
23S	830	0.04	23S	2029	0.04	23S	2445	0.04	23S	2577	0.04

Supplementary Table 3. Continued

23S	831	0.05	23S	2030	0.05	23S	2446	0.05	23S	2590	0.04
23S	832	0.05	23S	2031	0.06	23S	2447	0.08	uL3	Gln150	0.04
23S	833	0.04	23S	2032	0.08	23S	2448	0.16	uL16	Gly83	0.04
23S	860	0.04	23S	2033	0.07	23S	2449	0.21	uL20	Val30	0.04
23S	946	0.05	23S	2034	0.06	23S	2450	0.19			
23S	947	0.05	23S	2035	0.04	23S	2452	0.15			

Supplementary Table 4. Frequencies *f* of nodes in *k*-shortest pathways of the crystal structure 4v5h

	node	*f*		node	*f*		node	*F*		node	*f*
23S	**A2062**	20	23S	**U2586**	20	uL23	**Glu18**	20	uL23	**Glu18**	20
23S	**A2451**	20	23S	**A2451**	20	uL23	**Gln72**	20	uL22	**Gly91**	20
23S	C2063	14	23S	U2585	19	uL23	His15	20	23S	A1614	20
23S	G2061	9	PolyAla	Ala24	15	uL23	Lys33	20	uL23	Lys19	20
23S	A2450	8	P-tRNA	A76	11	uL23	Ser17	20	23S	A1392	18
23S	C2064	7	23S	C2063	10	uL23	Trp80	20	23S	C1314	16
PolyAla	Ala24	6	23S	A2450	9	uL23	Arg69	15	23S	C1315	16
P-tRNA	A76	5	PolyAla	Ala23	7	uL23	Lys66	15	23S	C1615	16
PolyAla	Ala21	4	23S	C2064	6	uL23	His70	14	23S	A1616	14
PolyAla	Ala22	4	23S	A2439	5	uL23	Lys68	14	23S	A1609	13
PolyAla	Ala23	4	23S	A2062	2	uL23	Val67	14	23S	U1316	12
23S	C2065	3	PolyAla	Ala21	2	uL23	Arg77	10	23S	G1332	12
23S	G2447	2	PolyAla	Ala22	2	uL23	Asp79	10	23S	U1313	10
23S	U2449	2	23S	G2061	1	uL23	Gly65	10	23S	A1610	7
23S	U2585	2				uL23	Lys64	10	23S	A1393	6
23S	G2446	1				uL23	Ser78	10	23S	C1611	6
23S	C2452	1				uL23	Val63	10	23S	C1612	6
23S	A2497	1				uL23	Ala13	6	23S	G1613	6
23S	C2501	1				uL23	Arg73	6	23S	G1317	4
23S	A2503	1				uL23	Arg76	6	23S	G1333	4
23S	U2504	1				uL23	Gly75	6	23S	G1334	4
						uL23	Ile74	6	23S	C1617	4
						uL23	Pro14	6	23S	G1331	3
						uL23	Val16	6	23S	C1335	3
						uL23	Leu32	5	23S	A1336	3
						uL23	Gly71	4	23S	U1318	1
									23S	C1319	1
									23S	G1324	1
									uL22	Arg92	1

Supplementary Table 5. Nucleotides/residues highly influencing A2062 in the simulations of *T. thermophilus* 70S

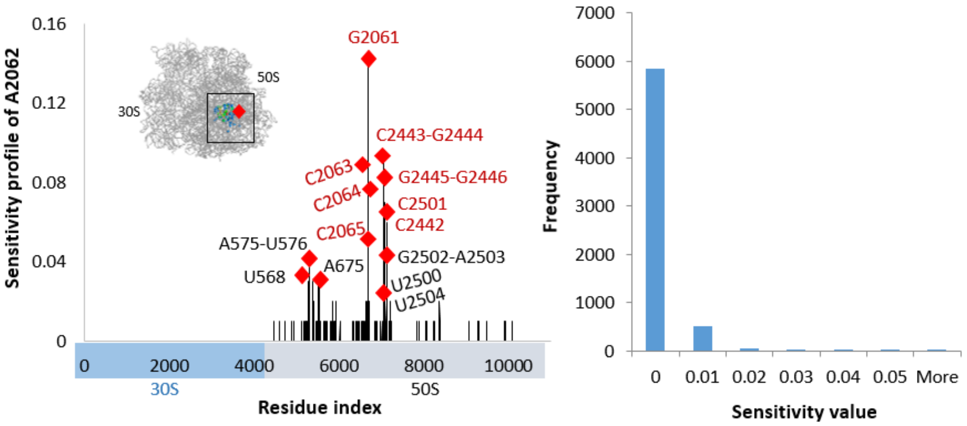

Residue		Sensitivity	Residue		Sensitivity	Residue		Sensitivity	Residue		Sensitivity
23S	563	0.02	23S	678	0.02	23S	2056	0.03	23S	2448	0.03
23S	564	0.02	23S	679	0.02	23S	2057	0.03	23S	2449	0.02
23S	565	0.02	23S	793	0.02	23S	2058	0.03	23S	2451	0.03
23S	566	0.02	23S	802	0.03	23S	2059	0.06	23S	2452	0.02
23S	567	0.02	23S	804	0.02	23S	2060	0.06	23S	2453	0.02
23S	568	0.03	23S	806	0.03	23S	2061	0.14	23S	2498	0.02
23S	569	0.02	23S	807	0.03	23S	2063	0.07	23S	2499	0.02
23S	572	0.02	23S	808	0.03	23S	2064	0.06	23S	2500	0.04
23S	574	0.02	23S	809	0.02	23S	2065	0.04	23S	2501	0.06
23S	575	0.03	23S	830	0.02	23S	2066	0.03	23S	2502	0.05
23S	576	0.04	23S	831	0.02	23S	2067	0.04	23S	2503	0.05
23S	577	0.03	23S	1190	0.02	23S	2068	0.03	23S	2504	0.04
23S	578	0.02	23S	1254	0.02	23S	2069	0.02	23S	2505	0.03
23S	579	0.02	23S	1255	0.02	23S	2070	0.02	23S	2586	0.02
23S	580	0.02	23S	1256	0.02	23S	2071	0.02	23S	2587	0.02
23S	581	0.02	23S	1257	0.02	23S	2072	0.02	uL4	67	0.02
23S	583	0.02	23S	1263	0.02	23S	2438	0.02	uL4	68	0.02
23S	585	0.02	23S	2016	0.02	23S	2439	0.02	uL4	69	0.02
23S	670	0.02	23S	2017	0.02	23S	2440	0.02	uL4	70	0.02
23S	671	0.02	23S	2019	0.02	23S	2441	0.04	uL4	71	0.02
23S	672	0.02	23S	2020	0.02	23S	2442	0.05	uL4	72	0.02
23S	673	0.02	23S	2034	0.02	23S	2443	0.08	uL4	73	0.02
23S	674	0.02	23S	2052	0.02	23S	2444	0.09	uL4	74	0.02
23S	675	0.03	23S	2053	0.02	23S	2445	0.07			
23S	676	0.02	23S	2054	0.03	23S	2446	0.07			
23S	677	0.02	23S	2055	0.03	23S	2447	0.04			

Supplementary Table 6. Nucleotides/residues highly influencing A2451 in the simulations of *T. thermophilus* 70S

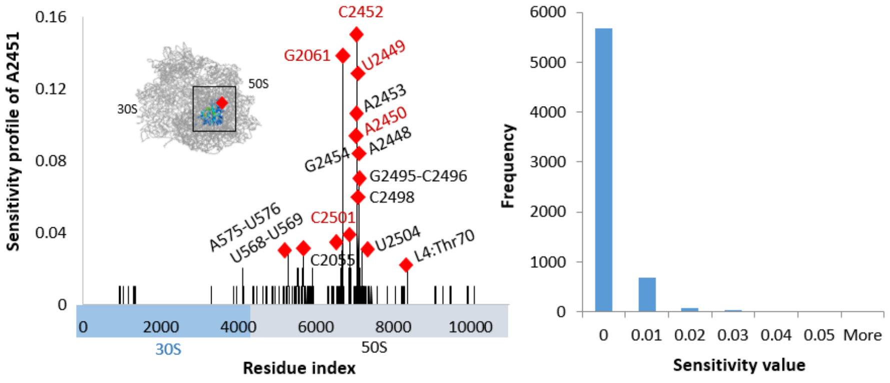

Residue		Sensitivity	Residue		Sensitivity	Residue		Sensitivity	Residue		Sensitivity
23S	562	0.02	23S	963	0.03	23S	2066	0.03	23S	2492	0.03
23S	564	0.02	23S	964	0.03	23S	2067	0.02	23S	2493	0.03
23S	565	0.02	23S	965	0.02	23S	2068	0.02	23S	2494	0.04
23S	566	0.02	23S	973	0.02	23S	2247	0.02	23S	2495	0.07
23S	567	0.02	23S	976	0.02	23S	2248	0.02	23S	2496	0.07
23S	568	0.03	23S	977	0.02	23S	2249	0.03	23S	2497	0.04
23S	569	0.03	23S	1255	0.02	23S	2250	0.03	23S	2498	0.07
23S	570	0.02	23S	2017	0.02	23S	2251	0.04	23S	2499	0.04
23S	571	0.02	23S	2026	0.02	23S	2252	0.03	23S	2500	0.06
23S	572	0.02	23S	2027	0.02	23S	2253	0.02	23S	2501	0.04
23S	573	0.02	23S	2029	0.02	23S	2265	0.02	23S	2502	0.02
23S	574	0.02	23S	2030	0.02	23S	2275	0.02	23S	2503	0.03
23S	575	0.03	23S	2031	0.02	23S	2276	0.02	23S	2504	0.04
23S	576	0.03	23S	2032	0.02	23S	2277	0.02	23S	2505	0.02
23S	577	0.02	23S	2033	0.02	23S	2443	0.02	23S	2506	0.02
23S	578	0.02	23S	2034	0.02	23S	2444	0.03	23S	2507	0.03
23S	806	0.02	23S	2052	0.02	23S	2445	0.03	23S	2508	0.02
23S	807	0.02	23S	2053	0.03	23S	2446	0.03	23S	2509	0.02
23S	808	0.02	23S	2054	0.02	23S	2447	0.03	23S	2512	0.02
23S	829	0.02	23S	2055	0.04	23S	2448	0.08	23S	2571	0.03
23S	830	0.02	23S	2056	0.02	23S	2449	0.12	23S	2572	0.02
23S	831	0.02	23S	2057	0.03	23S	2450	0.09	23S	2573	0.03
23S	832	0.02	23S	2059	0.03	23S	2452	0.15	23S	2574	0.02
23S	945	0.02	23S	2060	0.03	23S	2453	0.10	23S	2575	0.02
23S	946	0.02	23S	2061	0.14	23S	2454	0.06	uL4	70	0.02
23S	947	0.02	23S	2062	0.02	23S	2455	0.04	uL28	2	0.02
23S	948	0.02	23S	2063	0.02	23S	2456	0.03			
23S	956	0.02	23S	2064	0.03	23S	2457	0.03			
23S	962	0.03	23S	2065	0.03	23S	2491	0.02			

Supplementary Table 7. Frequencies *f* of nodes in *k*-shortest pathways of ClustENM conformers

	**node**	***f***		**node**	***f***		**node**	***f***		**node**	***f***
23S	**C2586**	2020	23S	**A2062**	2020	uL23	**Glu15**	2020	uL23	**Glu15**	2020
23S	**A2451**	2020	23S	**A2451**	2020	uL23	**Arg68**	2020	uL22	**Gly91**	2020
23S	C2440	1571	23S	A2450	1697	23S	G1338	1762	23S	A1614	2015
23S	A2587	1571	23S	C2063	1515	23S	G1337	1521	23S	G661	1558
23S	A2450	1554	23S	C2061	1414	23S	A1336	1521	uL23	Lys16	1307
23S	C2441	1492	23S	C2064	1151	uL23	Arg65	1521	23S	C1611	1153
23S	C2442	1391	23S	C2501	687	23S	U1335	1521	23S	C1613	1153
23S	C2064	1232	23S	C2065	687	uL23	Tyr69	1263	23S	C1612	1153
23S	C2443	1190	23S	G2251	465	uL23	Ser14	1231	23S	C1615	1109
23S	C2065	1110	23S	G2447	242	uL23	Leu66	1227	23S	A1610	1021
23S	G2444	921	23S	U2449	202	23S	G1334	976	23S	C1314	914
23S	C2066	902	23S	G2446	202	uL23	Gly67	858	23S	C1604	913
23S	G2445	703	23S	G2252	202	23S	G1339	857	23S	G1338	863
23S	G2067	700				uL23	Lys16	837	23S	A1616	863
23S	G2446	495				uL23	Arg73	349	23S	A1609	768
23S	G2069	482				uL23	Lys62	345	23S	C1605	744
23S	C2452	449				uL23	Lys64	291	uL23	Ser14	719
23S	U2506	449				23S	A64	288	23S	U1313	635
23S	U2584	449				uL23	Tys17	284	23S	A1603	634
23S	U2585	449				uL23	Leu70	157	23S	G1334	472
23S	G2583	355				uL23	Lys63	150	23S	A1336	472
23S	G2070	281				uL23	Arg76	124	23S	C1333	472
23S	C2507	254				uL23	Ala17	124	23S	G1337	472
23S	C2501	193				uL23	Trp29	123	23S	U1335	472
23S	G2447	192				uL23	Pro74	118	23S	G1606	426
23S	G2582	149				uL23	Phe28	76	23S	A1608	426
23S	G2508	99				uL23	Thr27	76	23S	G1332	373
23S	A2071	79				uL23	Val12	47	23S	C1617	291
23S	G2072	79				uL23	Gly61	34	23S	A1331	191
23S	G792	79				23S	U1312	30	23S	C1315	121
23S	G2061	67				uL23	Asp75	29	23S	G1310	101
uL4	Lys68	67				uL23	Lys72	28	23S	G1324	99
23S	C2063	50				uL23	Gly71	28	uL23	Tyr18	56
23S	G2251	22				uL23	Pro11	24	23S	G1309	18
23S	G2553	4				uL23	Ala10	24	uL22	Asp94	4
						23S	C1314	20	uL22	Ala93	4
						uL23	Leu13	10	uL22	Arg92	4
						23S	A1603	8	uL22	Leu86	4
						uL23	Val59	1	23S	G1325	4
						23S	U1341	1	23S	U1340	2
						uL23	Leu57	1	23S	A751	1
									23S	A789	1

Supplementary Table 8. Nucleotides/residues highly influencing U2586 in the simulations of *E. coli* 70S

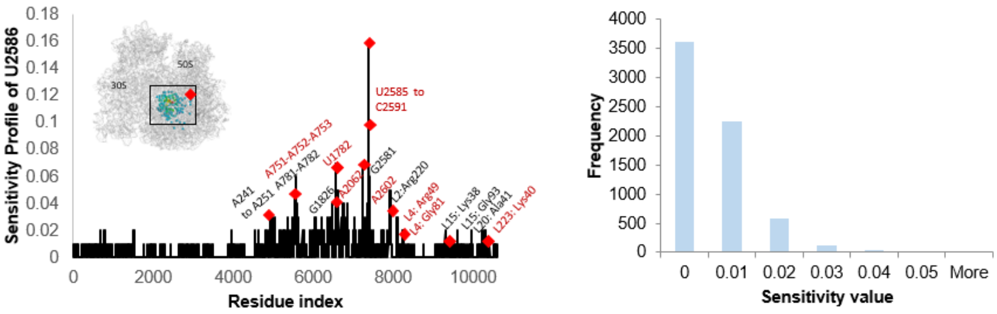

Residue		Sensitivity	Residue		Sensitivity	Residue		Sensitivity	Residue		Sensitivity
23S	739	0.03	23S	1619	0.03	23S	1938	0.04	23S	2550	0.03
23S	740	0.03	23S	1647	0.03	23S	1939	0.04	23S	2552	0.04
23S	741	0.04	23S	1658	0.03	23S	1940	0.03	23S	2553	0.03
23S	742	0.05	23S	1670	0.03	23S	1941	0.04	23S	2554	0.03
23S	743	0.04	23S	1671	0.03	23S	1956	0.04	23S	2578	0.03
23S	744	0.05	23S	1672	0.04	23S	1957	0.04	23S	2579	0.03
23S	748	0.03	23S	1673	0.04	23S	1968	0.03	23S	2580	0.03
23S	750	0.03	23S	1675	0.03	23S	1972	0.03	23S	2581	0.05
23S	752	0.04	23S	1773	0.04	23S	1973	0.04	23S	2582	0.06
23S	753	0.04	23S	1774	0.03	23S	1974	0.03	23S	2583	0.09
23S	754	0.04	23S	1775	0.03	23S	1975	0.03	23S	2584	0.1
23S	771	0.03	23S	1776	0.04	23S	1981	0.03	23S	2585	0.12
23S	772	0.03	23S	1777	0.02	23S	1983	0.04	23S	2587	0.16
23S	773	0.03	23S	1778	0.03	23S	1985	0.03	23S	2588	0.13
23S	778	0.03	23S	1779	0.04	23S	2012	0.03	23S	2589	0.1
23S	779	0.03	23S	1780	0.05	23S	2055	0.03	23S	2590	0.09
23S	780	0.03	23S	1781	0.04	23S	2056	0.03	23S	2591	0.05
23S	781	0.06	23S	1782	0.07	23S	2059	0.03	23S	2592	0.03
23S	782	0.04	23S	1783	0.04	23S	2063	0.03	23S	2593	0.03
23S	783	0.06	23S	1784	0.04	23S	2064	0.03	23S	2597	0.03
23S	784	0.06	23S	1785	0.04	23S	2068	0.03	23S	2598	0.05
23S	785	0.04	23S	1786	0.03	23S	2072	0.03	23S	2599	0.07
23S	786	0.03	23S	1787	0.04	23S	2073	0.03	23S	2600	0.06
23S	787	0.04	23S	1788	0.05	23S	2074	0.04	23S	2601	0.05
23S	788	0.03	23S	1789	0.03	23S	2075	0.04	23S	2602	0.04
23S	789	0.02	23S	1792	0.03	23S	2239	0.03	23S	2603	0.04
23S	790	0.03	23S	1804	0.03	23S	2240	0.03	23S	2604	0.06
23S	791	0.04	23S	1806	0.03	23S	2241	0.03	23S	2605	0.07
23S	792	0.04	23S	1814	0.03	23S	2438	0.03	23S	2606	0.04
23S	793	0.03	23S	1815	0.04	23S	2439	0.05	23S	2607	0.05

Supplementary Table 8. Continued

23S	794	0.03	23S	1816	0.03	23S	2440	0.07	23S	2608	0.06
23S	806	0.03	23S	1822	0.03	23S	2441	0.05	23S	2609	0.06
23S	807	0.03	23S	1823	0.03	23S	2443	0.03	23S	2610	0.03
23S	808	0.03	23S	1824	0.03	23S	2444	0.03	23S	2612	0.03
23S	831	0.03	23S	1825	0.04	23S	2449	0.03	uL3	131	0.03
23S	1257	0.03	23S	1826	0.05	23S	2503	0.03	uL3	136	0.03
23S	1264	0.03	23S	1827	0.04	23S	2504	0.03	uL3	137	0.03
23S	1428	0.03	23S	1828	0.03	23S	2508	0.03	uL3	139	0.03
23S	1560	0.03	23S	1903	0.03	23S	2509	0.03	uL3	141	0.03
23S	1615	0.03	23S	1937	0.03	23S	2511	0.03			

Supplementary Table 9. Nucleotides/residues highly influencing U2585 in the simulations of *E. coli* 70S

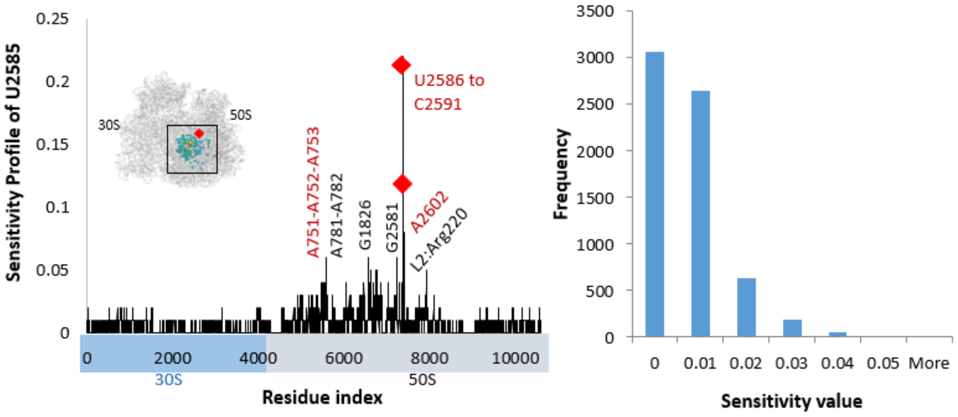

Residue	Sensitivity	Residue	Sensitivity	Residue	Sensitivity	Residue	Sensitivity
23S 126	0.03	23S 790	0.03	23S 1935	0.03	23S 2500	0.03
23S 193	0.03	23S 791	0.03	23S 1937	0.05	23S 2505	0.03
23S 194	0.03	23S 792	0.03	23S 1938	0.04	23S 2506	0.04
23S 198	0.03	23S 794	0.03	23S 1939	0.05	23S 2507	0.04
23S 199	0.03	23S 795	0.03	23S 1940	0.05	23S 2508	0.03
23S 249	0.03	23S 802	0.03	23S 1941	0.04	23S 2512	0.03
23S 250	0.03	23S 804	0.03	23S 1942	0.04	23S 2551	0.03
23S 251	0.03	23S 807	0.03	23S 1944	0.03	23S 2552	0.04
23S 381	0.03	23S 827	0.03	23S 1956	0.03	23S 2553	0.04
23S 448	0.03	23S 828	0.03	23S 1957	0.04	23S 2554	0.04
23S 449	0.03	23S 945	0.03	23S 1958	0.03	23S 2578	0.03
23S 467	0.03	23S 1256	0.04	23S 1965	0.04	23S 2579	0.03
23S 565	0.03	23S 1261	0.03	23S 1966	0.03	23S 2580	0.04
23S 577	0.03	23S 1328	0.03	23S 1967	0.03	23S 2581	0.05
23S 584	0.03	23S 1357	0.03	23S 1968	0.04	23S 2582	0.07
23S 585	0.03	23S 1375	0.03	23S 1969	0.03	23S 2583	0.11
23S 586	0.03	23S 1567	0.03	23S 1970	0.03	23S 2584	0.11
23S 587	0.03	23S 1568	0.03	23S 1971	0.03	23S 2586	0.22
23S 670	0.03	23S 1608	0.03	23S 1972	0.05	23S 2587	0.16
23S 671	0.03	23S 1617	0.03	23S 1973	0.03	23S 2588	0.11
23S 673	0.03	23S 1620	0.03	23S 1974	0.03	23S 2589	0.09
23S 674	0.04	23S 1648	0.03	23S 1981	0.04	23S 2590	0.06
23S 675	0.03	23S 1658	0.03	23S 1982	0.03	23S 2591	0.06
23S 676	0.03	23S 1659	0.03	23S 1983	0.03	23S 2592	0.05
23S 677	0.03	23S 1672	0.03	23S 1984	0.03	23S 2593	0.04
23S 679	0.03	23S 1673	0.04	23S 1985	0.03	23S 2594	0.03
23S 680	0.03	23S 1674	0.04	23S 1994	0.03	23S 2597	0.03
23S 682	0.03	23S 1677	0.03	23S 2017	0.03	23S 2598	0.04
23S 684	0.03	23S 1697	0.03	23S 2019	0.03	23S 2599	0.08

Supplementary Table 9. Continued

23S	687	0.03	23S	1773	0.04	23S	2031	0.03	23S	2600	0.05
23S	688	0.03	23S	1774	0.03	23S	2032	0.03	23S	2601	0.05
23S	691	0.03	23S	1775	0.04	23S	2033	0.03	23S	2602	0.06
23S	692	0.04	23S	1776	0.03	23S	2055	0.03	23S	2603	0.05
23S	693	0.04	23S	1778	0.03	23S	2056	0.03	23S	2604	0.08
23S	694	0.03	23S	1779	0.04	23S	2059	0.03	23S	2605	0.06
23S	729	0.03	23S	1780	0.04	23S	2060	0.03	23S	2606	0.08
23S	730	0.03	23S	1781	0.03	23S	2063	0.03	23S	2607	0.07
23S	731	0.03	23S	1782	0.04	23S	2064	0.04	23S	2608	0.05
23S	732	0.03	23S	1783	0.03	23S	2065	0.03	23S	2609	0.05
23S	738	0.03	23S	1784	0.04	23S	2066	0.03	23S	2610	0.03
23S	740	0.03	23S	1785	0.05	23S	2068	0.03	23S	2611	0.03
23S	741	0.03	23S	1786	0.04	23S	2069	0.03	uL2	50	0.03
23S	742	0.03	23S	1787	0.04	23S	2071	0.03	uL2	51	0.03
23S	744	0.04	23S	1788	0.06	23S	2072	0.03	uL2	53	0.03
23S	748	0.03	23S	1789	0.05	23S	2073	0.04	uL2	55	0.03
23S	750	0.03	23S	1790	0.03	23S	2074	0.04	uL2	214	0.03
23S	751	0.03	23S	1791	0.03	23S	2075	0.04	uL2	215	0.03
23S	752	0.03	23S	1795	0.03	23S	2076	0.03	uL2	220	0.04
23S	753	0.03	23S	1804	0.03	23S	2085	0.03	uL2	221	0.04
23S	754	0.03	23S	1805	0.03	23S	2229	0.03	uL2	222	0.05
23S	759	0.03	23S	1806	0.03	23S	2239	0.04	uL2	223	0.04
23S	760	0.03	23S	1807	0.03	23S	2240	0.04	uL2	224	0.04
23S	761	0.03	23S	1812	0.03	23S	2242	0.03	uL2	225	0.05
23S	763	0.03	23S	1813	0.03	23S	2251	0.03	uL2	226	0.04
23S	764	0.03	23S	1814	0.04	23S	2393	0.03	uL2	228	0.03
23S	765	0.03	23S	1815	0.03	23S	2427	0.03	uL2	229	0.03
23S	767	0.03	23S	1816	0.03	23S	2429	0.03	uL2	230	0.03
23S	770	0.03	23S	1819	0.03	23S	2430	0.03	uL2	231	0.03
23S	777	0.04	23S	1821	0.03	23S	2438	0.03	uL2	232	0.03
23S	778	0.03	23S	1822	0.03	23S	2439	0.04	uL2	233	0.03
23S	779	0.03	23S	1823	0.03	23S	2440	0.06	uL2	234	0.03
23S	780	0.03	23S	1824	0.04	23S	2441	0.04	uL2	235	0.03
23S	781	0.04	23S	1825	0.04	23S	2442	0.04	uL2	237	0.03
23S	782	0.04	23S	1826	0.05	23S	2444	0.03	uL2	239	0.03
23S	783	0.06	23S	1827	0.05	23S	2448	0.03	uL2	240	0.03
23S	784	0.05	23S	1828	0.04	23S	2450	0.04	uL2	243	0.03
23S	785	0.05	23S	1829	0.03	23S	2451	0.03	uL2	244	0.03
23S	786	0.03	23S	1830	0.03	23S	2454	0.03	uL3	133	0.03
23S	787	0.03	23S	1842	0.03	23S	2498	0.03			
23S	788	0.03	23S	1903	0.04	23S	2499	0.03			

Supplementary Table 10. Nucleotides/residues highly influencing U2585 in the simulations of *T. thermophilus* 70S

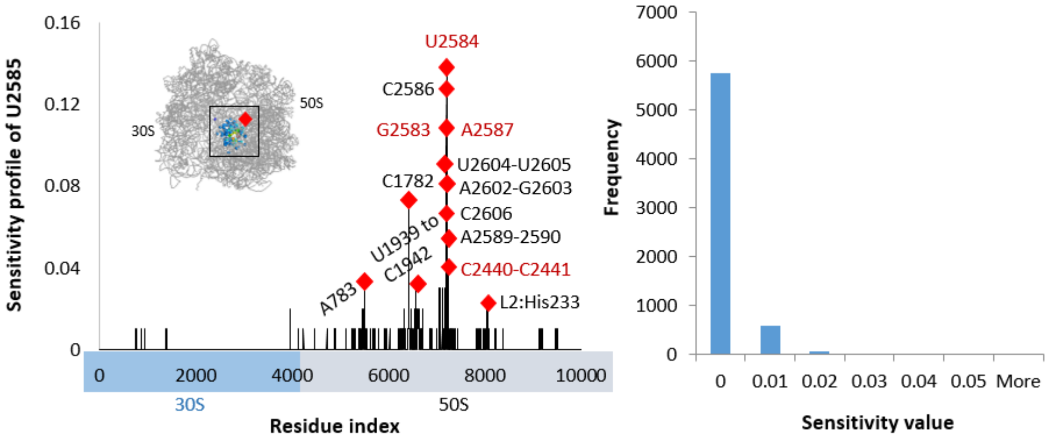

Residue		Sensitivity	Residue		Sensitivity	Residue		Sensitivity	Residue		Sensitivity
23S	740	0.02	23S	1824	0.02	23S	1984	0.02	23S	2591	0.03
23S	741	0.02	23S	1825	0.02	23S	1985	0.02	23S	2592	0.02
23S	742	0.02	23S	1826	0.02	23S	1995	0.02	23S	2593	0.02
23S	752	0.02	23S	1827	0.02	23S	2073	0.02	23S	2594	0.02
23S	753	0.02	23S	1902	0.02	23S	2438	0.03	23S	2598	0.03
23S	781	0.02	23S	1936	0.02	23S	2439	0.03	23S	2599	0.03
23S	782	0.02	23S	1937	0.02	23S	2440	0.03	23S	2600	0.03
23S	783	0.03	23S	1938	0.02	23S	2441	0.03	23S	2601	0.05
23S	784	0.02	23S	1939	0.03	23S	2442	0.02	23S	2602	0.07
23S	785	0.02	23S	1940	0.03	23S	2452	0.02	23S	2603	0.06
23S	786	0.02	23S	1941	0.03	23S	2507	0.03	23S	2604	0.08
23S	1672	0.02	23S	1942	0.03	23S	2508	0.02	23S	2605	0.08
23S	1673	0.02	23S	1944	0.02	23S	2550	0.02	23S	2606	0.06
23S	1674	0.02	23S	1945	0.02	23S	2551	0.02	23S	2607	0.03
23S	1675	0.02	23S	1956	0.02	23S	2552	0.03	23S	2608	0.04
23S	1769	0.02	23S	1957	0.02	23S	2553	0.02	23S	2609	0.03
23S	1770	0.02	23S	1958	0.02	23S	2554	0.02	uL2	223	0.02
23S	1775	0.02	23S	1965	0.02	23S	2571	0.02	uL2	224	0.02
23S	1780	0.03	23S	1967	0.02	23S	2580	0.02	uL2	225	0.02
23S	1781	0.02	23S	1968	0.02	23S	2581	0.03	uL2	226	0.02
23S	1782	0.07	23S	1969	0.02	23S	2582	0.06	uL2	227	0.02
23S	1783	0.02	23S	1971	0.02	23S	2583	0.10	uL2	233	0.02
23S	1784	0.02	23S	1972	0.02	23S	2584	0.14	uL2	234	0.02
23S	1785	0.02	23S	1973	0.02	23S	2586	0.13	uL2	235	0.02
23S	1786	0.02	23S	1974	0.02	23S	2587	0.10	uL2	236	0.02
23S	1787	0.02	23S	1975	0.02	23S	2588	0.07	uL2	237	0.02
23S	1788	0.03	23S	1982	0.02	23S	2589	0.05	uL2	238	0.02
23S	1789	0.02	23S	1983	0.02	23S	2590	0.04			

Supplementary Table 11. Nucleotides/residues highly influencing C2586 in the simulations of *T. thermophilus* 70S

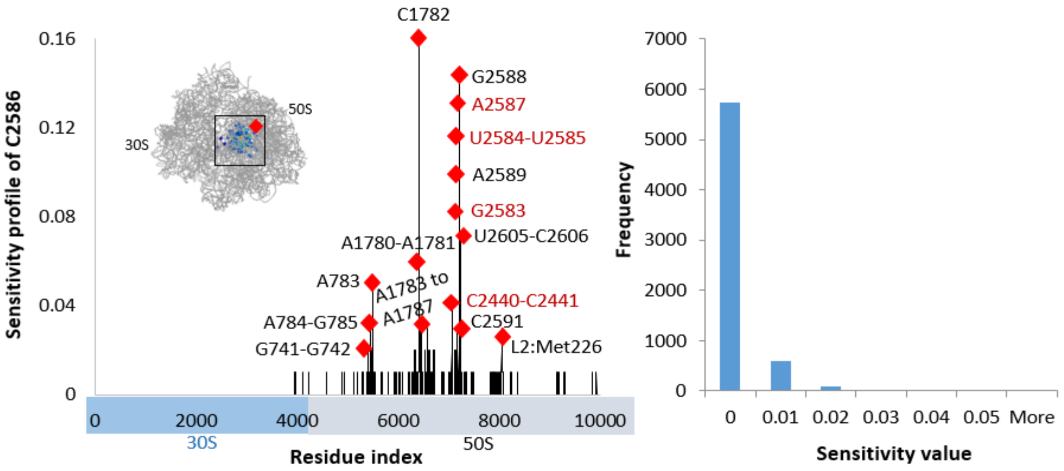

Residue		Sensitivity	Residue		Sensitivity	Residue		Sensitivity	Residue		Sensitivity
23S	689	0.02	23S	1786	0.02	23S	2068	0.02	23S	2593	0.02
23S	740	0.02	23S	1787	0.03	23S	2070	0.02	23S	2594	0.02
23S	741	0.03	23S	1788	0.03	23S	2073	0.02	23S	2595	0.02
23S	742	0.03	23S	1789	0.02	23S	2074	0.02	23S	2597	0.02
23S	744	0.02	23S	1791	0.02	23S	2075	0.02	23S	2598	0.03
23S	752	0.02	23S	1824	0.02	23S	2437	0.02	23S	2599	0.03
23S	753	0.02	23S	1825	0.02	23S	2438	0.03	23S	2600	0.03
23S	754	0.02	23S	1826	0.03	23S	2439	0.03	23S	2601	0.05
23S	759	0.02	23S	1827	0.03	23S	2440	0.04	23S	2602	0.04
23S	764	0.02	23S	1828	0.02	23S	2441	0.04	23S	2603	0.04
23S	780	0.02	23S	1901	0.02	23S	2442	0.02	23S	2604	0.05
23S	781	0.03	23S	1902	0.02	23S	2443	0.02	23S	2605	0.06
23S	782	0.03	23S	1903	0.02	23S	2444	0.02	23S	2606	0.07
23S	783	0.05	23S	1937	0.02	23S	2445	0.02	23S	2607	0.03
23S	784	0.04	23S	1938	0.02	23S	2446	0.02	23S	2608	0.06
23S	785	0.04	23S	1939	0.03	23S	2500	0.02	23S	2609	0.02
23S	786	0.02	23S	1940	0.03	23S	2507	0.02	uL2	222	0.02
23S	787	0.02	23S	1941	0.02	23S	2508	0.02	L2	223	0.02
23S	1673	0.02	23S	1942	0.02	23S	2550	0.02	L2	224	0.02
23S	1674	0.02	23S	1944	0.02	23S	2551	0.02	uL2	225	0.02
23S	1770	0.02	23S	1945	0.02	23S	2552	0.03	uL2	226	0.03
23S	1773	0.02	23S	1956	0.02	23S	2553	0.02	uL2	227	0.02
23S	1774	0.02	23S	1957	0.02	23S	2554	0.02	uL2	231	0.02
23S	1775	0.02	23S	1971	0.02	23S	2581	0.02	uL2	232	0.02
23S	1776	0.02	23S	1972	0.02	23S	2582	0.03	uL2	233	0.02
23S	1777	0.02	23S	1974	0.02	23S	2583	0.06	uL2	234	0.03
23S	1778	0.02	23S	1982	0.02	23S	2584	0.11	uL2	235	0.02
23S	1779	0.02	23S	1983	0.02	23S	2585	0.1	uL2	236	0.02

Supplementary Table 11. Continued

23S	1780	0.05	23S	1984	0.02	23S	2587	0.13	uL2	237	0.02
23S	1781	0.05	23S	1985	0.02	23S	2588	0.14	uL2	238	0.02
23S	1782	0.16	23S	1986	0.02	23S	2589	0.09	uL2	239	0.02
23S	1783	0.03	23S	2063	0.02	23S	2590	0.05	uL2	240	0.02
23S	1784	0.03	23S	2064	0.02	23S	2591	0.04	uL2	242	0.02
23S	1785	0.03	23S	2067	0.02	23S	2592	0.02			

Supplementary Table 12. Nucleotides/residues highly influencing Glu18 (uL23) in the simulations of *E. coli* 70S

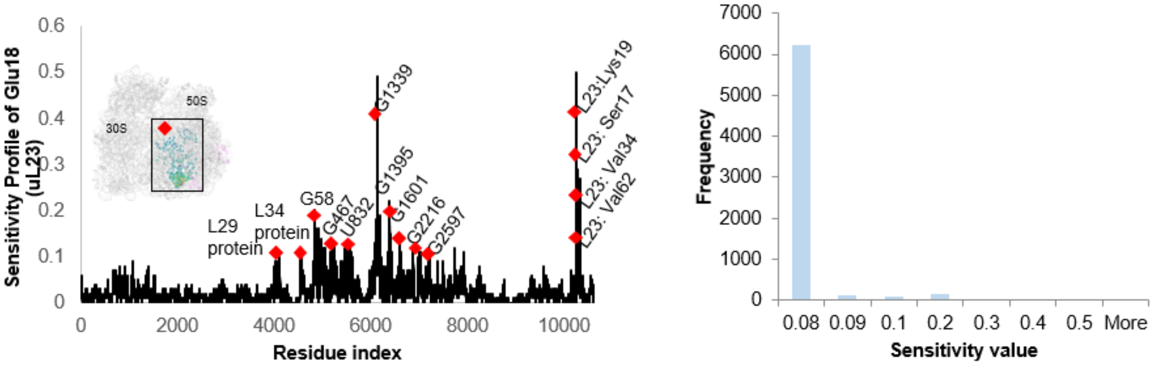

Residue		Sensitivity	Residue		Sensitivity	Residue		Sensitivity	Residue		Sensitivity
23S	51	0.10	23S	398	0.13	23S	1392	0.18	23S	2240	0.11
23S	52	0.10	23S	452	0.12	23S	1393	0.19	23S	2243	0.10
23S	53	0.09	23S	458	0.10	23S	1394	0.12	23S	2597	0.08
23S	54	0.11	23S	459	0.13	23S	1395	0.16	23S	2598	0.09
23S	55	0.12	23S	460	0.10	23S	1396	0.13	uL23	7	0.14
23S	56	0.15	23S	467	0.11	23S	1397	0.12	uL23	8	0.13
23S	57	0.17	23S	682	0.10	23S	1398	0.18	uL23	9	0.16
23S	58	0.20	23S	683	0.12	23S	1399	0.19	uL23	10	0.18
23S	59	0.19	23S	693	0.12	23S	1598	0.15	uL23	11	0.20
23S	60	0.16	23S	694	0.10	23S	1599	0.15	uL23	12	0.21
23S	61	0.14	23S	695	0.10	23S	1600	0.22	uL23	13	0.14
23S	62	0.15	23S	763	0.12	23S	1601	0.20	uL23	14	0.25
23S	63	0.14	23S	764	0.12	23S	1602	0.18	uL23	15	0.24
23S	64	0.14	23S	769	0.11	23S	1603	0.17	uL23	16	0.29
23S	65	0.12	23S	770	0.10	23S	1604	0.18	uL23	17	0.38
23S	66	0.09	23S	771	0.10	23S	1605	0.11	uL23	19	0.50
23S	67	0.09	23S	772	0.13	23S	1606	0.10	uL23	20	0.38
23S	68	0.10	23S	773	0.10	23S	1607	0.09	uL23	21	0.39
23S	69	0.13	23S	775	0.09	23S	1608	0.09	uL23	22	0.32
23S	70	0.13	23S	776	0.09	23S	1609	0.09	uL23	23	0.21
23S	71	0.13	23S	777	0.11	23S	1610	0.08	uL23	24	0.22
23S	72	0.14	23S	778	0.09	23S	1611	0.09	uL23	25	0.19
23S	73	0.16	23S	779	0.12	23S	1612	0.09	uL23	26	0.09
23S	74	0.14	23S	794	0.11	23S	1613	0.09	uL23	27	0.08
23S	75	0.10	23S	832	0.10	23S	1614	0.07	uL23	28	0.10
23S	76	0.10	23S	1305	0.12	23S	1615	0.11	uL23	29	0.13
23S	114	0.12	23S	1306	0.13	23S	1616	0.09	uL23	30	0.17
23S	115	0.12	23S	1307	0.12	23S	1617	0.15	uL23	31	0.22
23S	116	0.14	23S	1308	0.11	23S	1644	0.11	uL23	32	0.27
23S	117	0.15	23S	1309	0.09	23S	1800	0.12	uL23	33	0.29
23S	118	0.16	23S	1310	0.11	23S	1801	0.09	uL23	34	0.28

23S	119	0.16	23S	1311	0.11	23S	1802	0.10	uL23	35	0.24
23S	120	0.16	23S	1312	0.15	23S	1803	0.08	uL23	36	0.21
23S	121	0.14	23S	1313	0.18	23S	1804	0.08	uL23	37	0.17
23S	122	0.10	23S	1314	0.15	23S	1805	0.10	uL23	38	0.14
23S	123	0.10	23S	1315	0.17	23S	1806	0.08	uL23	39	0.15
23S	124	0.10	23S	1316	0.12	23S	1807	0.11	uL23	40	0.16
23S	125	0.14	23S	1317	0.11	23S	1808	0.08	uL23	41	0.13
23S	126	0.15	23S	1331	0.12	23S	1809	0.07	uL23	42	0.14
23S	127	0.14	23S	1332	0.15	23S	1810	0.09	uL23	43	0.16
23S	128	0.15	23S	1333	0.13	23S	1811	0.08	uL23	44	0.15
23S	129	0.13	23S	1334	0.13	23S	1812	0.10	uL23	45	0.13
23S	130	0.14	23S	1335	0.14	23S	1813	0.12	uL23	46	0.13
23S	149	0.12	23S	1336	0.19	23S	1814	0.09	uL23	47	0.12
23S	150	0.12	23S	1337	0.16	23S	1815	0.10	uL23	48	0.09
23S	178	0.11	23S	1338	0.34	23S	1816	0.10	uL23	57	0.13
23S	179	0.14	23S	1339	0.49	23S	1817	0.13	uL23	58	0.15
23S	180	0.14	23S	1340	0.38	23S	1818	0.10	uL23	59	0.17
23S	181	0.12	23S	1341	0.28	23S	1819	0.09	uL23	60	0.19
23S	182	0.11	23S	1342	0.24	23S	1820	0.08	uL23	61	0.15
23S	251	0.12	23S	1343	0.21	23S	1821	0.08	uL23	62	0.15
23S	252	0.12	23S	1344	0.17	23S	1822	0.10	uL23	63	0.19
23S	375	0.09	23S	1345	0.11	23S	1823	0.08	uL23	64	0.12
23S	376	0.09	23S	1346	0.15	23S	1824	0.10	uL23	65	0.11
23S	377	0.08	23S	1347	0.11	23S	1825	0.09	uL23	79	0.10
23S	378	0.10	23S	1348	0.09	23S	1826	0.11	uL23	80	0.14
23S	379	0.08	23S	1349	0.16	23S	2072	0.10	uL23	81	0.23
23S	380	0.09	23S	1350	0.15	23S	2074	0.12	uL23	82	0.27
23S	381	0.10	23S	1371	0.13	23S	2075	0.10	uL23	83	0.23
23S	382	0.11	23S	1372	0.11	23S	2203	0.11	uL23	84	0.26
23S	383	0.10	23S	1373	0.12	23S	2214	0.10	uL23	85	0.22
23S	393	0.10	23S	1374	0.10	23S	2216	0.11	uL23	86	0.17
23S	394	0.09	23S	1375	0.13	23S	2232	0.10	uL23	87	0.13
23S	395	0.09	23S	1376	0.12	23S	2234	0.10			
23S	396	0.10	23S	1377	0.12	23S	2238	0.09			
23S	397	0.09	23S	1378	0.10	23S	2239	0.10			

Supplementary Table 13. Nucleotides/residues highly influencing Glu15 (uL23) in the simulations of *T. thermophilus* 70S

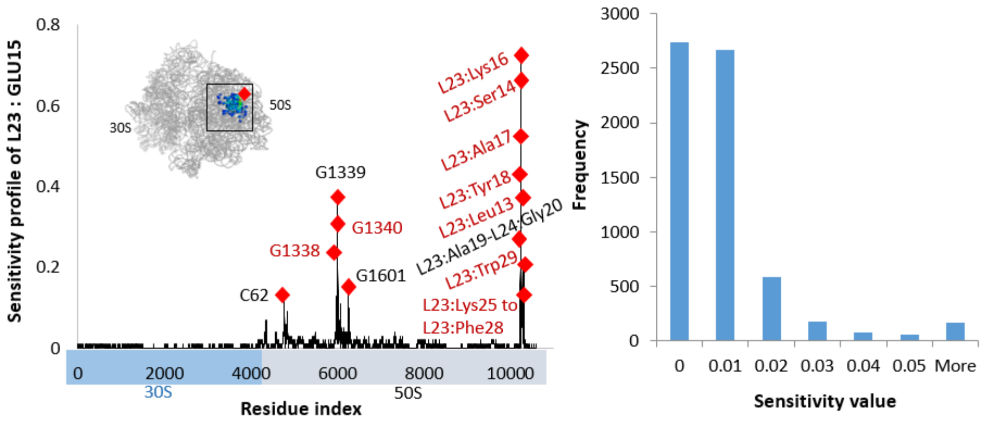

Residue		Sensitivity	Residue		Sensitivity	Residue		Sensitivity	Residue		Sensitivity
23S	51	0.06	23S	1338	0.20	uL23	9	0.22	uL23	55	0.12
23S	56	0.06	23S	1339	0.37	uL23	10	0.20	uL23	56	0.12
23S	57	0.07	23S	1340	0.31	uL23	11	0.29	uL23	57	0.11
23S	58	0.12	23S	1341	0.18	uL23	12	0.27	uL23	58	0.13
23S	59	0.13	23S	1342	0.20	uL23	13	0.37	uL23	59	0.12
23S	60	0.07	23S	1343	0.14	uL23	14	0.66	uL23	60	0.08
23S	61	0.08	23S	1344	0.09	uL23	16	0.72	uL23	61	0.06
23S	62	0.11	23S	1345	0.07	uL23	17	0.46	uL23	62	0.07
23S	63	0.07	23S	1346	0.08	uL23	18	0.38	uL23	63	0.07
23S	64	0.08	23S	1347	0.06	uL23	19	0.29	uL23	72	0.07
23S	65	0.08	23S	1348	0.06	uL23	20	0.29	uL23	73	0.08
23S	69	0.06	23S	1349	0.06	uL23	21	0.22	uL23	74	0.07
23S	70	0.07	23S	1351	0.06	uL23	22	0.12	uL23	75	0.09
23S	71	0.06	23S	1386	0.07	uL23	23	0.16	uL23	76	0.11
23S	72	0.09	23S	1387	0.07	uL23	24	0.18	uL23	77	0.18
23S	83	0.09	23S	1389	0.06	uL23	25	0.24	uL23	78	0.20
23S	74	0.08	23S	1392	0.06	uL23	26	0.23	uL23	79	0.19
23S	76	0.06	23S	1393	0.07	uL23	27	0.24	uL23	80	0.19
23S	113	0.06	23S	1394	0.07	uL23	28	0.25	uL23	81	0.20
23S	114	0.06	23S	1395	0.11	uL23	29	0.27	uL23	82	0.19
23S	115	0.06	23S	1396	0.07	uL23	30	0.22	uL23	83	0.17
23S	116	0.06	23S	1397	0.09	uL23	31	0.20	uL23	84	0.10
23S	118	0.06	23S	1398	0.11	uL23	32	0.14	uL23	85	0.07
23S	128	0.07	23S	1399	0.10	uL23	33	0.11	uL23	87	0.08
23S	129	0.08	23S	1400	0.06	uL23	34	0.13	uL23	88	0.06
23S	130	0.07	23S	1404	0.07	uL23	35	0.13	uL29	32	0.06
23S	131	0.08	23S	1406	0.06	uL23	36	0.12	uL29	33	0.06
23S	142A	0.07	23S	1596	0.09	uL23	37	0.12	uL29	35	0.06
23S	143	0.09	23S	1597	0.11	uL23	38	0.13	uL29	36	0.06

23S	143A	0.08	23S	1598	0.13	uL23	39	0.15	uL29	38	0.06
23S	144	0.08	23S	1599	0.13	uL23	40	0.13	uL29	39	0.07
23S	145	0.06	23S	1600	0.15	uL23	41	0.11	uL29	52	0.06
23S	1312	0.13	23S	1601	0.14	uL23	42	0.11	uL29	53	0.06
23S	1313	0.10	23S	1602	0.09	uL23	43	0.12	uL29	54	0.07
23S	1314	0.13	23S	1603	0.10	uL23	44	0.11	uL29	55	0.06
23S	1315	0.09	23S	1604	0.10	uL23	45	0.06	uL29	56	0.06
23S	1316	0.07	23S	1605	0.06	uL23	48	0.06	uL29	57	0.06
23S	1317	0.06	uL23	3	0.06	uL23	49	0.07	uL29	58	0.06
23S	1333	0.06	uL23	4	0.08	uL23	50	0.10	uL29	59	0.06
23S	1334	0.07	uL23	5	0.12	uL23	51	0.12	uL29	60	0.06
23S	1335	0.10	uL23	6	0.16	uL23	52	0.12			
23S	1336	0.11	uL23	7	0.20	uL23	53	0.13			
23S	1337	0.12	uL23	8	0.22	uL23	54	0.14			

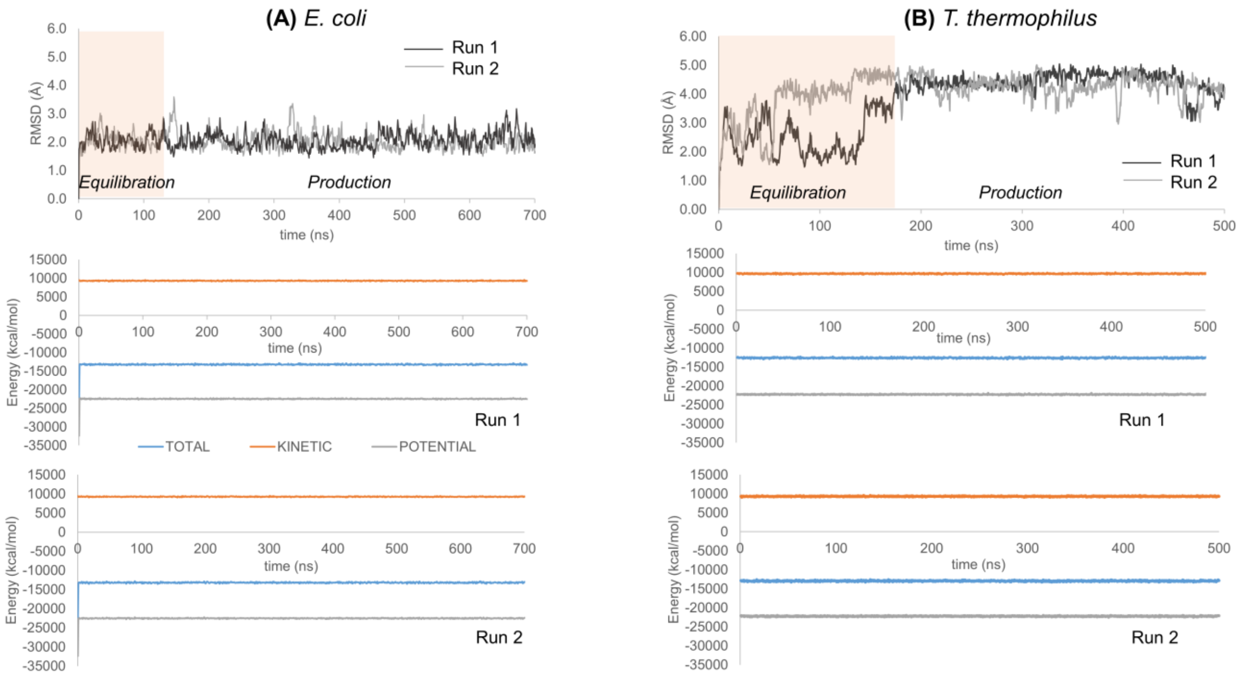

Supplementary Figure 1. Root mean square deviation and energy profiles for the CGMD simulations of **(A)** *E. coli* (PDB ID: 4v5h) and **(B)** *T. thermophilus* (PDB ID: 4v5d).

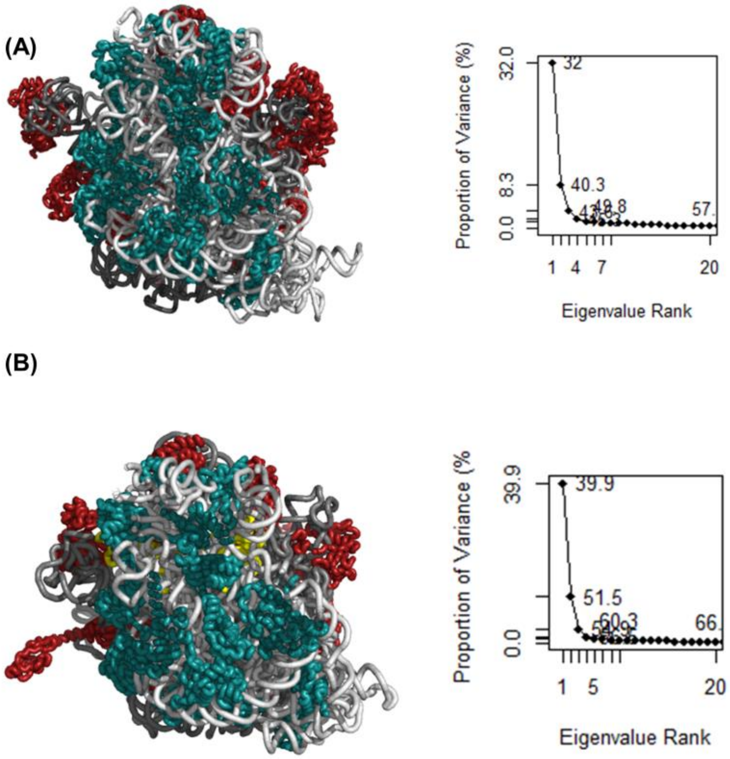

Supplementary Figure 2. Scree plots for the principal component analyses of the CGMD simulations of **(A)** *E. coli* (PDB ID: 4v5h) and **(B)** *T. thermophilus* (PDB ID: 4v5d). The small subunit is colored light grey (rRNA) and teal (r-proteins), the large subunit is shown in dark grey (rRNAs) and red (r-proteins).

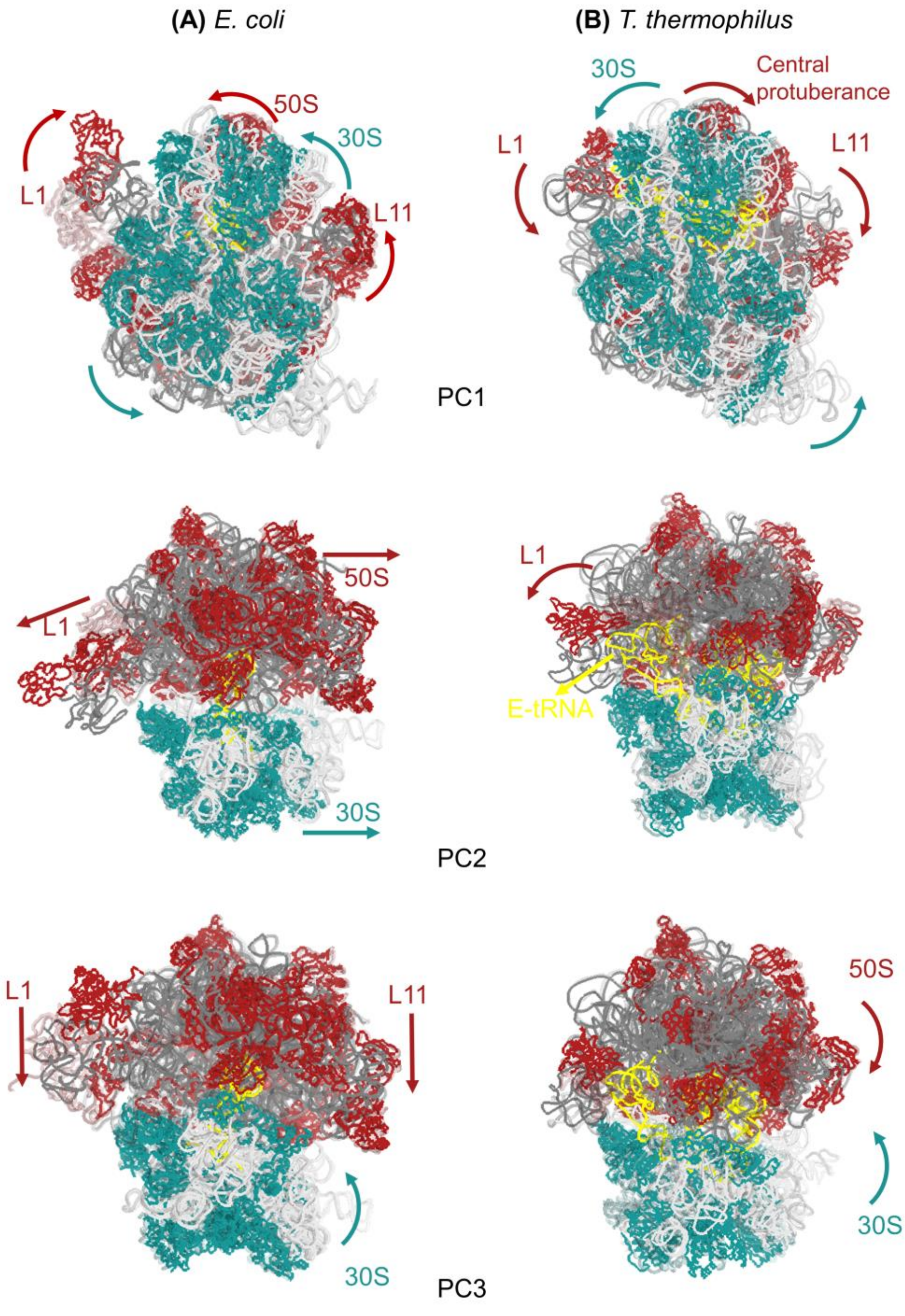

Supplementary Figure 3. First three principal components from **(A)** *E. coli* (PDB ID: 4v5h) and **(B)** *T. thermophilus* (PDB ID: 4v5d) CGMD simulations. In **(B)**, flexible bL9 is excluded from the analysis of *T. thermophilus* simulations to clearly observe the collective motions. Conformational changes are exaggerated to clearly present the findings. One conformer is transparent, where the directions of motion of the structural components are indicated in arrows. The small subunit is colored light grey (rRNA) and teal (r-proteins), the large subunit is shown in dark grey (rRNAs) and red (r-proteins).

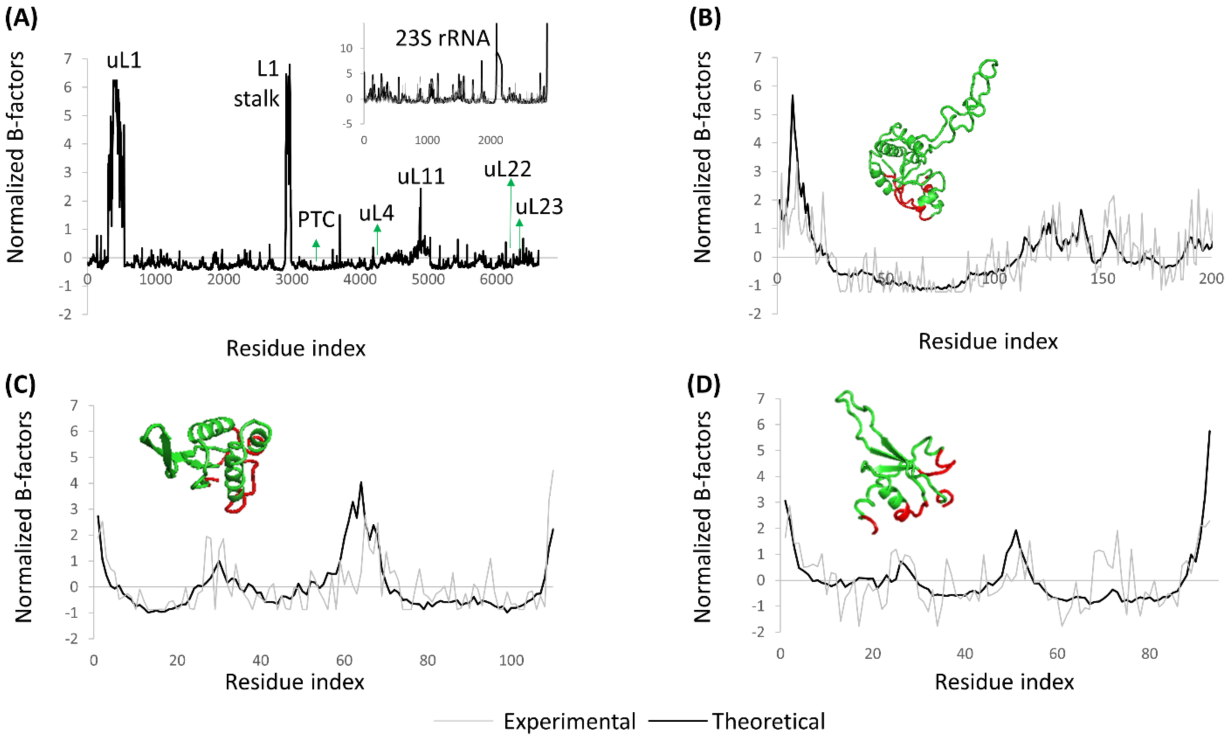

Supplementary Figure 4. Experimental and theoretical normalized B-factors of *E. coli* **(A)** large subunit 50S nucleotides/residues, **(B)** uL4, **(C)** uL22, and **(D)** uL23. In **(A)**, the locations of uL1, PTC, uL4, uL22, uL23 are indicated. For clarity, comparison of the experimental and theoretical normalized B-factors is given in inset only for 23S rRNA. In **(B)**, **(C)** and **(D)**, the most flexible parts of uL4, uL22 and uL23 indicated in red.

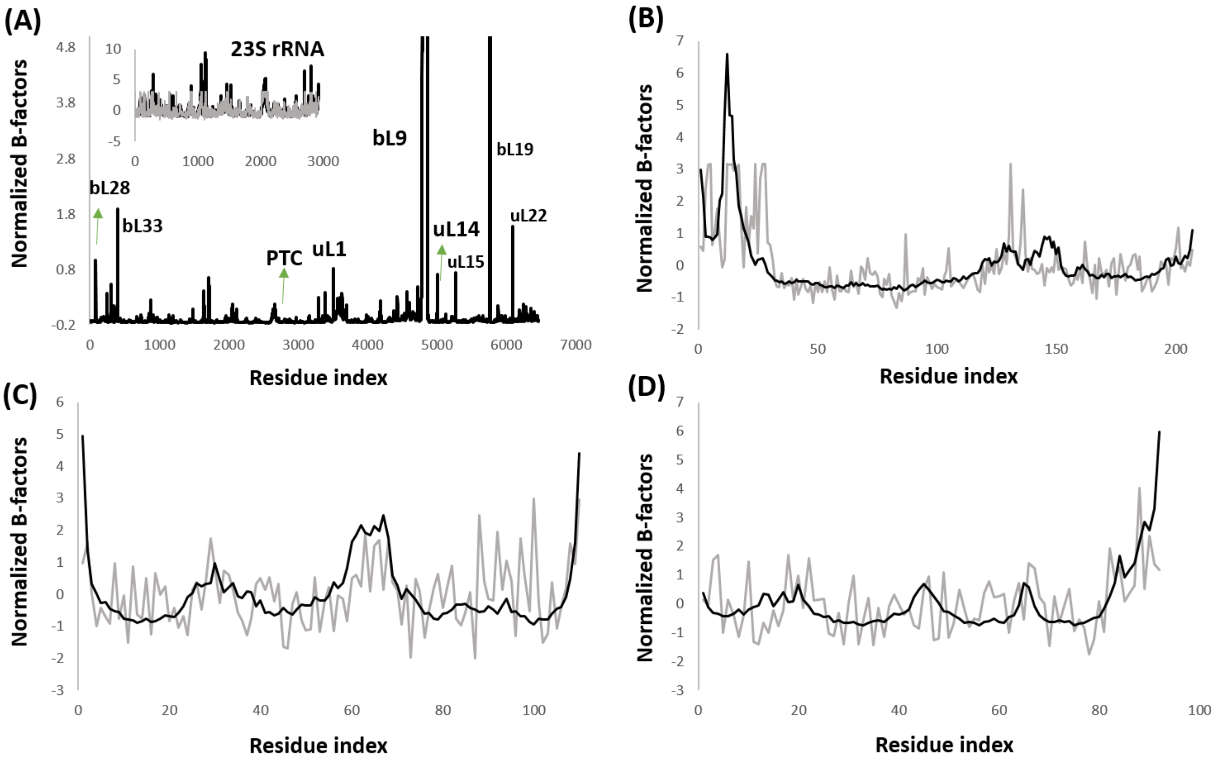

Supplementary Figure 5. Experimental and theoretical normalized B-factors of *T. thermophilus* **(A)** large subunit 50S nucleotides/residues, **(B)** uL4, **(C)** uL22, and **(D)** uL23. In **(A)**, the locations of uL1, PTC, uL4, uL22, uL23 are indicated. For clarity, comparison of the experimental and theoretical normalized B-factors is given in inset only for 23S rRNA.

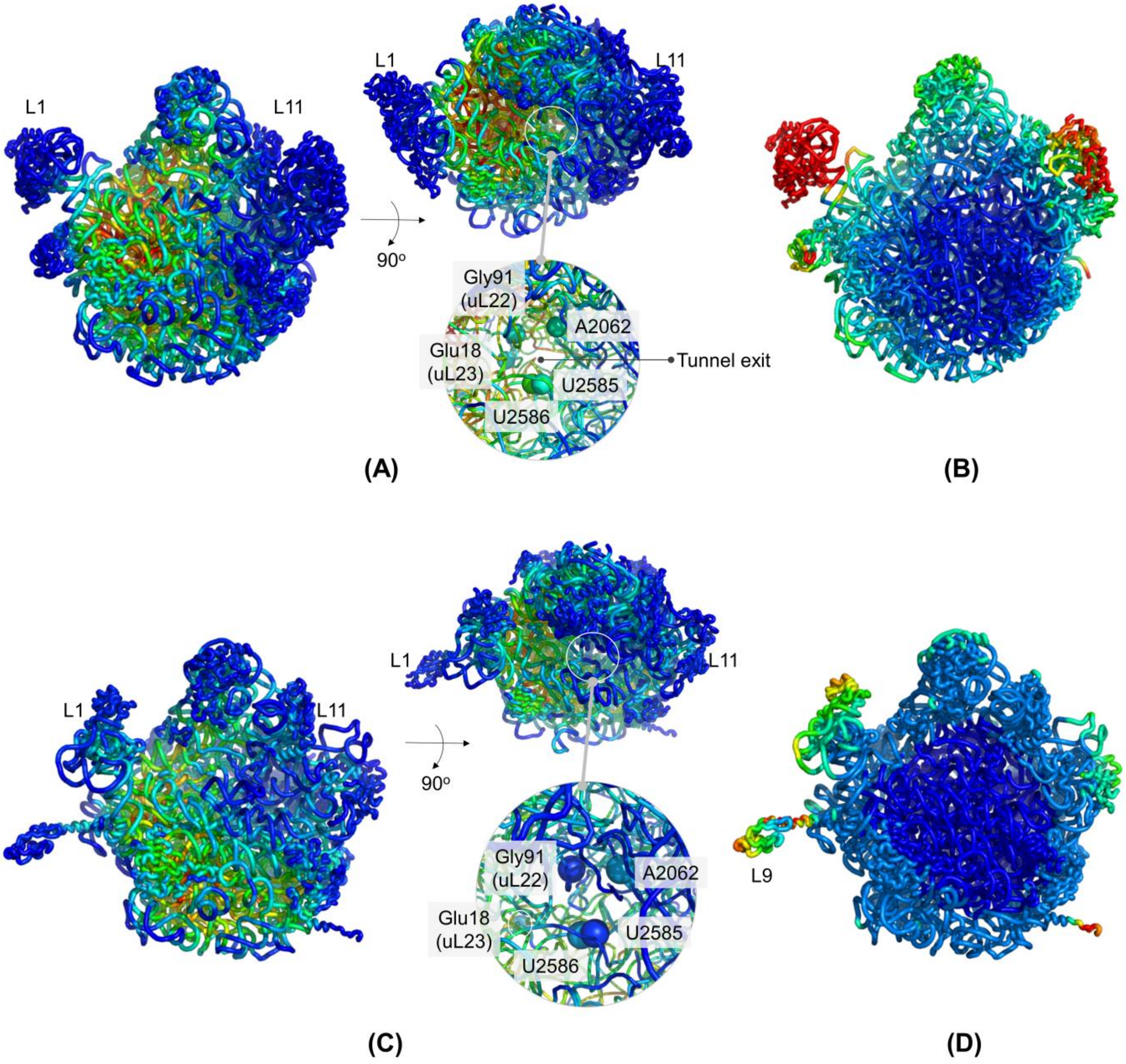

Supplementary Figure 6. (A) Effectors and **(B)** sensors in *E. coli*, and **(C)** effectors and **(D)** sensors in *T. thermophilus* large subunit 50S. In **(A)** and **(C)**, red to dark blue demonstrates decreasing ability of being an effector in the whole structure. The critical functional nucleotides/residues investigated in this study are labeled and shown in spheres. In **(B)** and **(D)**, red regions indicate the most sensitive parts to a perturbation, while dark blue regions point to most insensitive regions when the whole structure is considered.

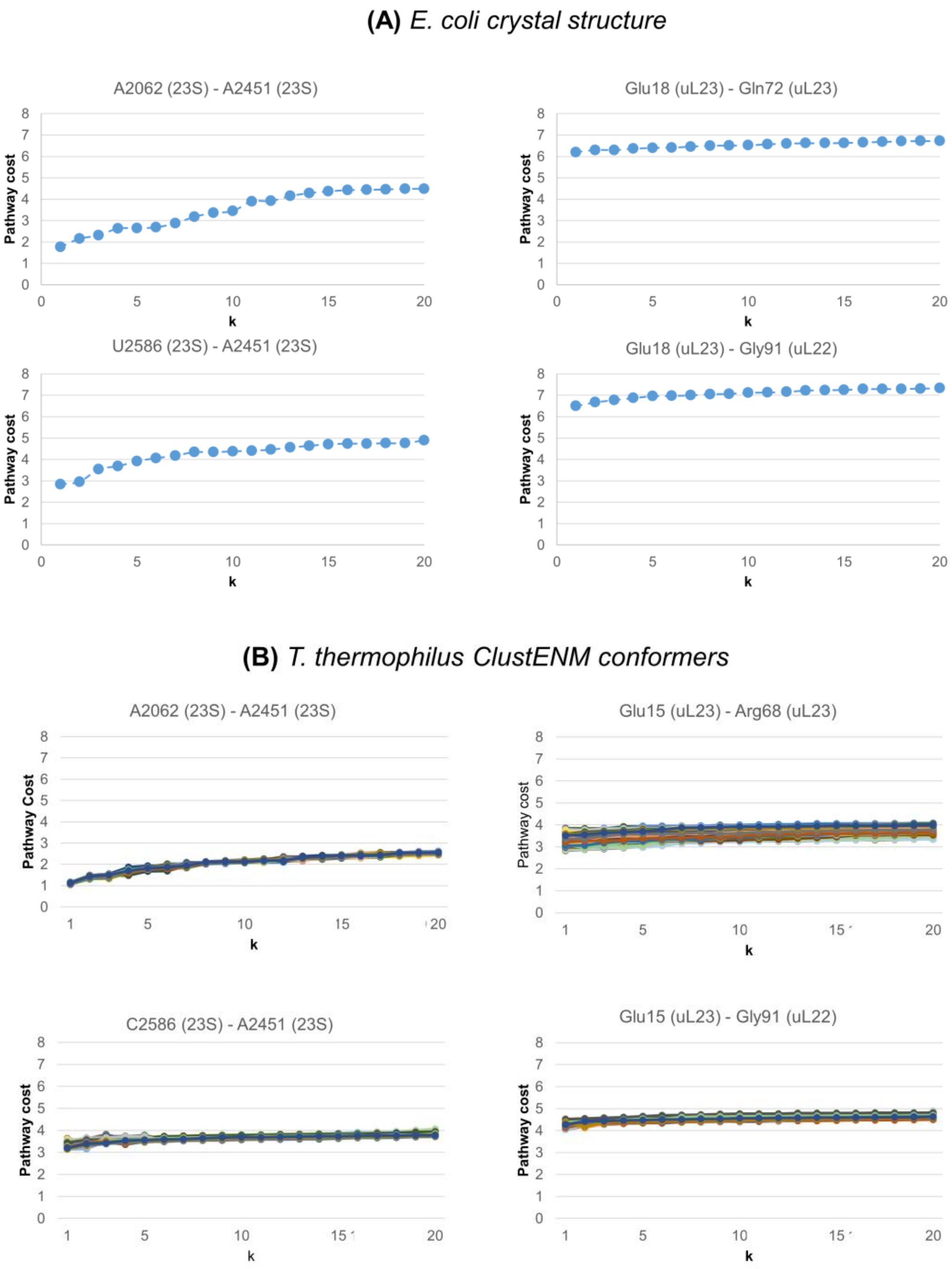

Supplementary Figure 7. k *vs* pathways costs for **(A)** *E. coli* crystal structure and **(B)** 101 *T. thermophilus* 50S conformers. Costs of pathways for the same source-sink pairs are different for **(A)** and **(B)** since ClustENM conformers are energetically minimized.

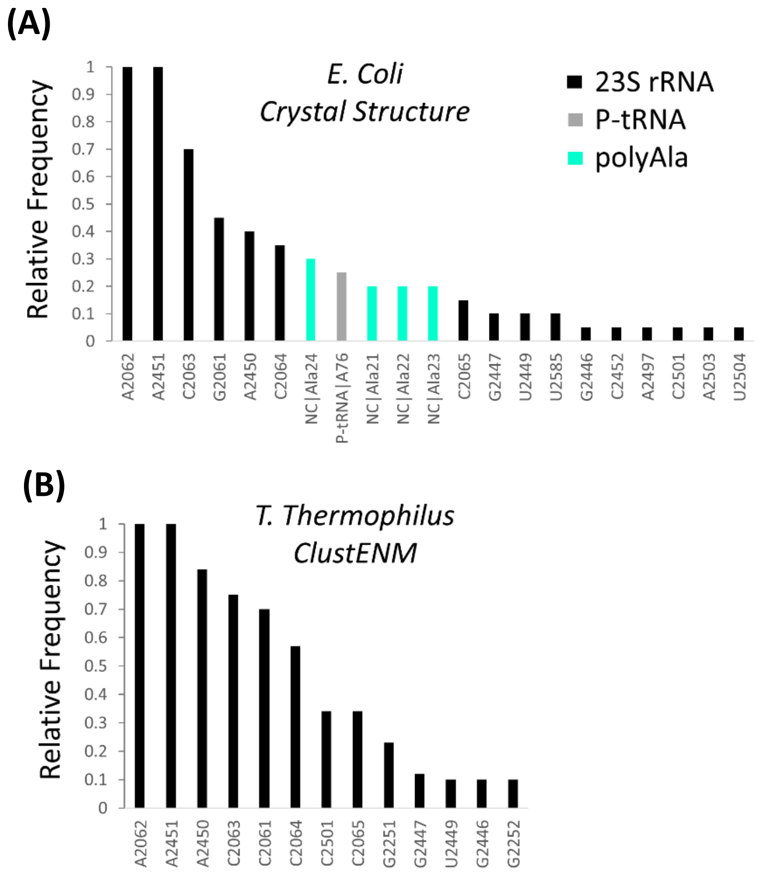

Supplementary Figure 8. Relative frequencies of nucleotides/residues on the *k*-shortest pathways from A2062 to A2451 on **(A)** *E. coli* large subunit 50S (PDB ID 4v5h), and **(B)** ClustENM conformers of *T. thermophilus* generated from large subunit 50S (PDB ID 4v9m)

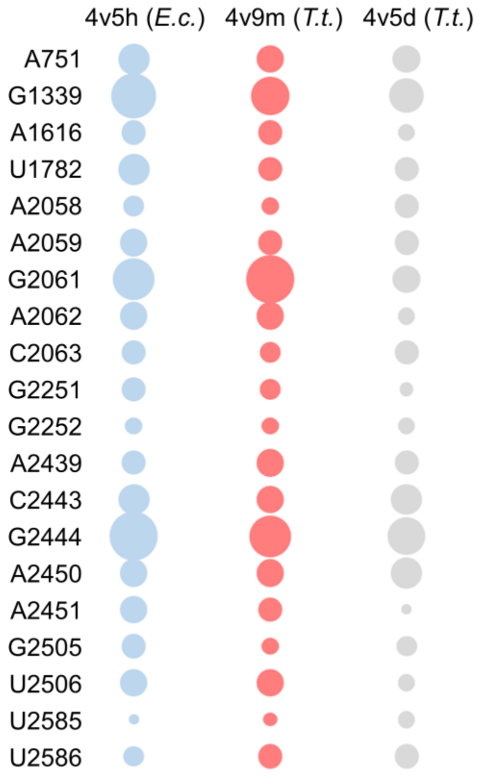

Supplementary Figure 9. Number of contacts of functionally important nucleotides on 23S rRNA. Here, tRNAs and polypeptide chain in the tunnel are excluded. Bubble scaling is from 3 to 14 contacts. Two nucleotides are considered as in contact if they have heavy atom-atom pairs within a cutoff distance of 4.5 Å, including van der Waals interactions.

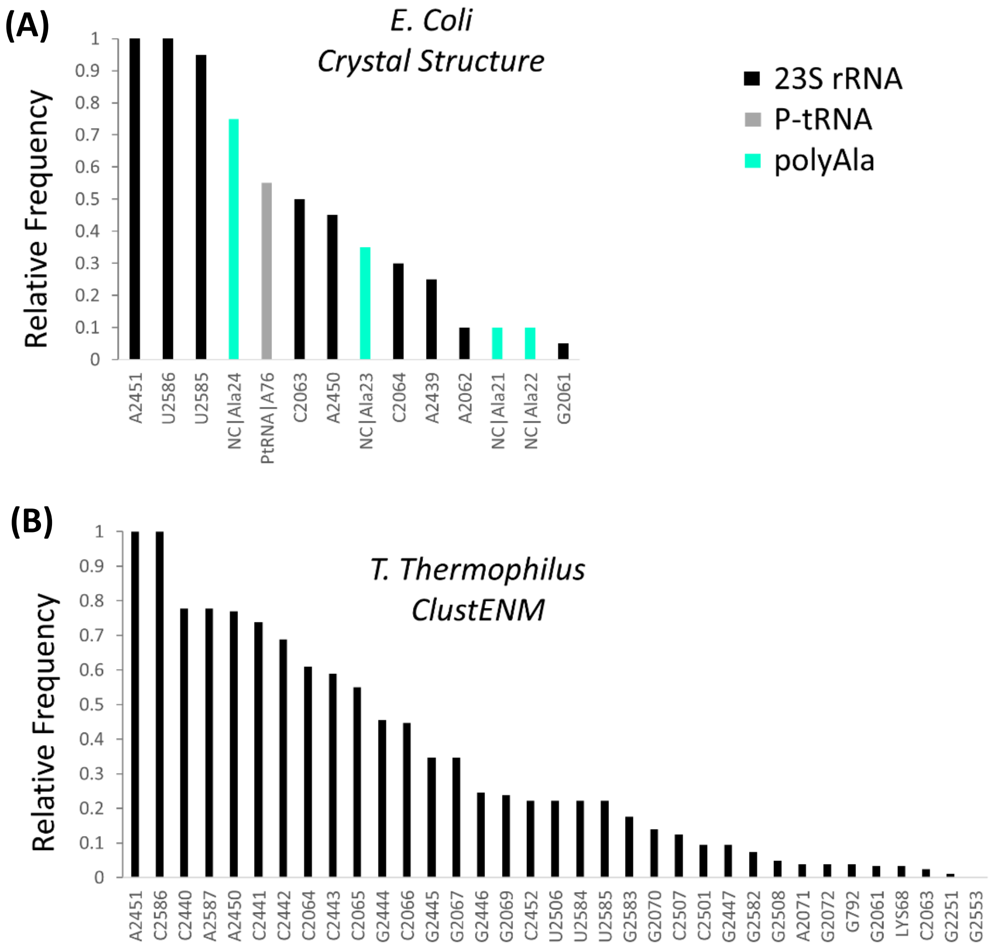

Supplementary Figure 10. Relative frequencies of nucleotides/residues on the *k*-shortest pathways from U2586 to A2451 on **(A)** *E. coli* large subunit 50S (PDB ID 4v5h), and **(B)** ClustENM conformers of *T. thermophilus* generated from large subunit 50S (PDB ID 4v9m)

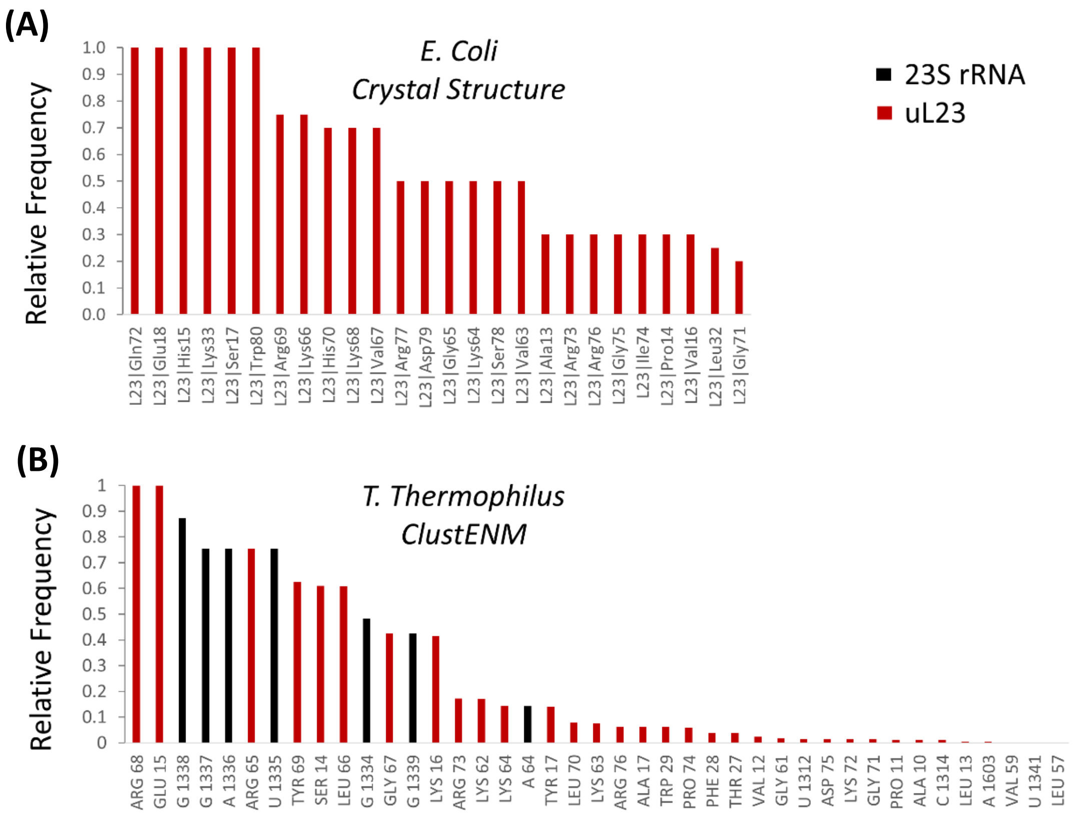

Supplementary Figure 11. Relative frequencies of nucleotides/residues on the *k*-shortest pathways from **(A)** Glu18 (uL23) to Gln72 (uL23) on *E. coli* large subunit 50S (PDB ID 4v5h), and **(B)** from Glu15 (uL23) to Arg68 (uL23) on ClustENM conformers of *T. thermophilus* generated from large subunit 50S (PDB ID 4v9m)

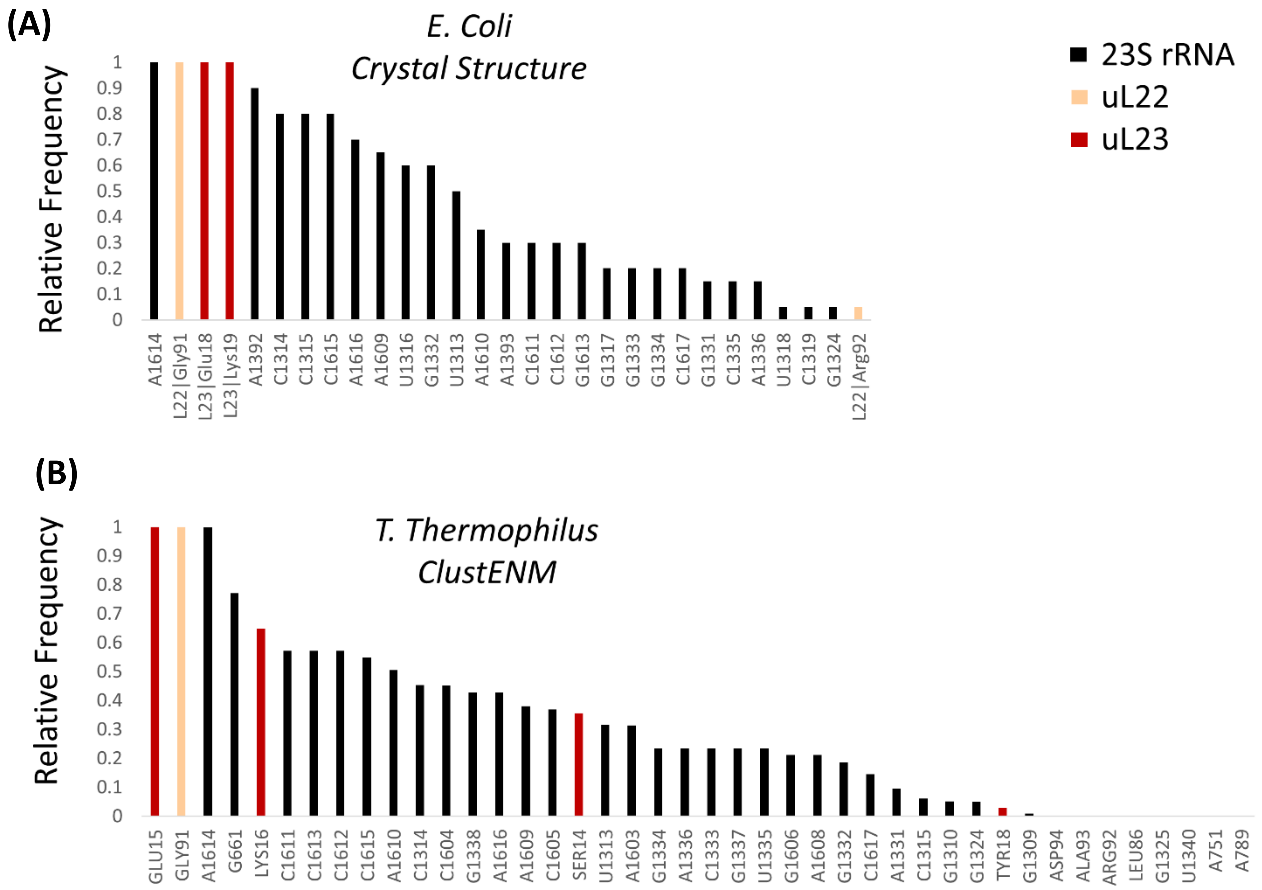

Supplementary Figure 12. Relative frequencies of nucleotides/residues on the *k*-shortest pathways from **(A)** Glu18 (uL23) to Gly91 (uL22) on *E. coli* large subunit 50S (PDB ID 4v5h), and **(B)** from Glu15 (uL23) to Gly91 (uL22) on ClustENM conformers of *T. thermophilus* generated from large subunit 50S (PDB ID 4v9m)

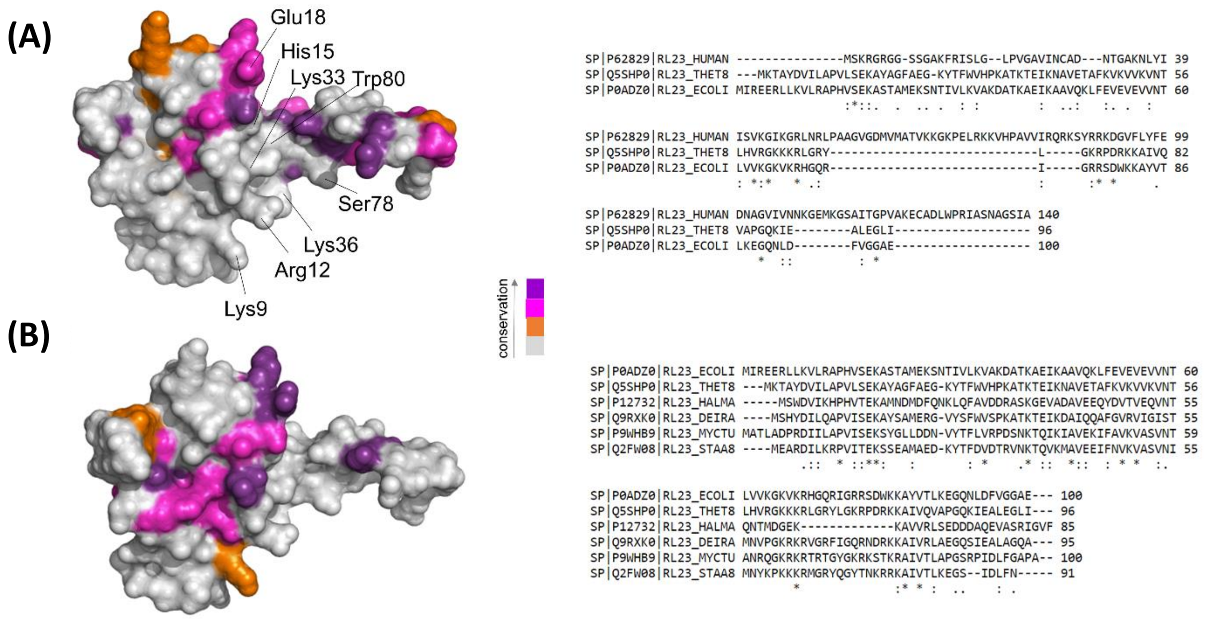

Supplementary Figure 13. Multiple sequence alignment for uL23 of **(A)** *E. coli*, *T. thermophilus* and *H. Sapiens*, **(B)** *E. coli*, *T. thermophilus*, *H. marismortui*, *D. radiodurans*, *M. tuberculosis* and *S. aureus*. Purple to grey coloring on *E. coli* uL23 structure indicates decrease in conservation. Chaperone binding site is shown by Glu18.
